# Cathepsin B-Oriented Screening, Isolation, and Antitumor Validation of Bioactive Metabolites from *Sargassum polycystum*

**DOI:** 10.3390/md24070231

**Published:** 2026-07-01

**Authors:** Wanchao Hou, Lingqiu Zhang, Kai Yu, Jinhua Lu, Congyao Qin, Minmin Qin, Xiuqing Xu, Zhengcai Du, Erwei Hao, Jiagang Deng, Xiaotao Hou

**Affiliations:** 1 Guangxi Key Laboratory of Efficacy Study on Chinese Materia Medica, Guangxi University of Chinese Medicine, Nanning 530200, China; houwc@gxtcmu.edu.cn (W.H.); lingqiuz11@outlook.com (L.Z.); bioneer@foxmail.com (K.Y.); jinhualu_1998@163.com (J.L.); qincongyao@163.com (C.Q.); 19955316329@163.com (M.Q.); 18778110759@163.com (X.X.); duzhengcai8@163.com (Z.D.); haoew@gxtcmu.edu.cn (E.H.); 2 University Engineering Research Center of Reutilization of Traditional Chinese Medicine Resources, Guangxi University of Chinese Medicine, Nanning 530200, China; 3 Guangxi Key Laboratory of TCM Formulas Theory and Transformation for Damp Diseases, Guangxi University of Chinese Medicine, Nanning 530200, China; 4 School of Pharmacy, Guangxi University of Chinese Medicine, Nanning 530200, China

**Keywords:** *Sargassum polycystum*, marine medicinal algae, cathepsin B, non-small cell lung cancer, target-guided screening, antitumor metabolites

## Abstract

Marine medicinal algae represent a valuable reservoir of bioactive metabolites for anticancer drug discovery, yet the efficient identification of target-relevant compounds from chemically complex marine matrices remains challenging. In this study, an integrated cathepsin B-oriented strategy was developed to discover, prioritize, isolate, and validate antitumor metabolites from the brown alga *Sargassum polycystum*. Affinity ultrafiltration LC-MS was first applied to screen CTSB-binding constituents from the crude extract, followed by molecular docking, molecular dynamics simulation, and gray relational analysis for multidimensional candidate prioritization. Seven CTSB-binding metabolites were characterized, including chlorogenic acid, caffeic acid, cynarin, loliolide, taxifolin, senkyunolide H, and dihydroactinidiolide, with binding degrees of 73.99–85.61% at 2.5 U/mL CTSB. Molecular docking showed predicted binding affinities ranging from −6.3 to −9.4 kcal/mol, compared with −10.2 kcal/mol for the positive control CA-074Me. Integrated computational and biological evaluation identified caffeic acid, cynarin, and taxifolin as the top-ranked candidates. Preparative recovery was then achieved using counter-current chromatography combined with semi-preparative HPLC, and the isolated compounds were structurally identified by LC-MS/MS and NMR. Cellular assays in NCI-H1975 cells suggested that these metabolites reduced CTSB-associated enzymatic activity and intracellular CTSB-related fluorescence signals to different extents, with phenolic acid-type compounds exhibiting comparatively stronger effects. At the extract level, *S. polycystum* dose-dependently suppressed NCI-H1975 xenograft tumor growth, with inhibition rates of 48.78%, 36.58%, and 22.86% in the high-, middle-, and low-dose groups, respectively, without evident hepatorenal histopathological toxicity. This effect was associated with reduced CTSB, Ki-67, and Bcl-2 staining, increased Bax staining, enhanced apoptosis, and ultrastructural alterations in tumor tissues. Overall, this study provides a practical CTSB-oriented workflow for discovering antitumor metabolites from marine medicinal algae and supports further investigation of *S. polycystum* as a potential source of anti-NSCLC candidates.

## 1. Introduction

*Sargassum* spp. are brown macroalgae belonging to the phylum Phaeophyta, order Fucales, and family *Sargassaceae*. They are widely distributed in temperate and tropical marine regions and are commonly found along the coasts of China, where they mainly grow on rocks in the middle and lower intertidal zones [1]. As important marine medicinal algae, *Sargassum* species have attracted increasing attention as natural sources of structurally diverse bioactive metabolites, including polyphenols, flavonoids, lactones, polysaccharides, and other functional constituents. In traditional Chinese medicine, *Sargassum* has a long history of medicinal use. As early as two thousand years ago, Shennong Bencao Jing recorded its effects in treating goiter-like masses and dispersing nodular qi stagnation, and the Chinese Pharmacopoeia lists it as a commonly used medicinal material with the functions of resolving phlegm, softening hard masses, promoting diuresis, and alleviating edema. Despite this long-standing medicinal relevance, the biological activities and active constituents of *Sargassum* remain insufficiently explored [2,3]. Among these species, *Sargassum polycystum* (*S. polycystum*) is a representative brown alga traditionally used for the treatment of goiter, lymph node tuberculosis, edema, and inflammatory disorders [4,5]. Modern studies have further shown that *S. polycystum* contains abundant bioactive metabolites and exhibits multiple biological effects, including antioxidant, anti-inflammatory, and antitumor activities [6]. Therefore, *S. polycystum* represents a valuable marine algal material for the discovery of bioactive compounds.

Non-small cell lung cancer (NSCLC) accounts for approximately 80–85% of all lung cancer cases and remains one of the leading causes of cancer-related mortality worldwide. Although therapeutic strategies such as platinum-based chemotherapy, EGFR tyrosine kinase inhibitors, ALK inhibitors, anti-VEGF antibodies, immune checkpoint inhibitors, and multitargeted agents have improved clinical outcomes, their long-term efficacy is still limited by drug resistance, systemic toxicity, tumor heterogeneity, and poor patient tolerance [7,8]. Therefore, the discovery of bioactive compounds with multi-level antitumor effects remains an important direction for NSCLC-related drug research. Marine medicinal resources, including algae used in traditional Chinese medicine, are particularly attractive because they contain structurally diverse metabolites that may act on multiple biological processes involved in tumor progression [9,10].

Cathepsin B (CTSB), a lysosomal cysteine protease, was selected as the primary target in this study because of its close involvement in tumor progression and its biological relevance to NSCLC. Under physiological conditions, CTSB participates in lysosomal protein turnover and intracellular homeostasis. In tumor cells, however, aberrant CTSB expression, secretion, or relocalization has been associated with extracellular matrix degradation, protease network activation, invasion, metastasis, angiogenesis, and apoptosis regulation [11,12,13]. CTSB can act intracellularly through lysosome-related pathways and can also be secreted into the extracellular space, where it contributes to matrix remodeling and tumor cell invasion. These biological functions make CTSB a relevant proteolytic target for evaluating compounds that may interfere with tumor-supporting processes [14].

In NSCLC, elevated CTSB expression has been reported in tumor tissues compared with adjacent or normal lung tissues, and higher CTSB levels have been associated with advanced TNM stage, lymph node metastasis, invasive phenotypes, and poor clinicopathological characteristics. Experimental studies have also indicated that CTSB is involved in lung cancer cell migration, invasion, and metastasis-related processes, suggesting that CTSB is not only a tumor-associated protease, but also a functionally relevant molecule in NSCLC progression [15]. In addition, because CTSB possesses a defined catalytic active site and can be evaluated using enzymatic, cellular, and molecular assays, it has been considered a feasible target for inhibitor discovery and target-oriented screening. Nevertheless, the role of CTSB in tumor biology is context-dependent, and CTSB modulation may influence multiple processes, including invasion, proliferation, lysosomal function, and apoptosis-related signaling. Therefore, CTSB is suitable for the target-guided discovery of antitumor candidates, but the biological consequences of CTSB modulation require integrated validation at the molecular, cellular, and in vivo levels [16,17].

Previous studies have shown that natural products can regulate CTSB or CTSB-related proteolytic pathways. Several plant-derived phenolic acids, flavonoids, alkaloids, terpenoids, and polyphenolic compounds have been reported to affect tumor invasion, apoptosis, lysosomal function, and matrix degradation partly through the modulation of cathepsins, matrix metalloproteinases, or related protease networks [18]. Marine-derived metabolites, including sulfated polysaccharides, phlorotannins, polyphenols, and other algal constituents, have also been reported to suppress tumor progression by regulating apoptosis, oxidative stress, inflammatory signaling, extracellular matrix remodeling, and protease-associated pathways [19,20,21]. However, most previous studies on *Sargassum* and *S. polycystum* have focused on crude extracts or polysaccharide fractions, especially fucoidan, whereas systematic CTSB-oriented screening of small-molecule metabolites from *S. polycystum* remains limited. This gap restricts the understanding of the small-molecule material basis underlying the antitumor effects of this marine medicinal alga.

In target-oriented natural product research, two major challenges remain: the rapid identification of target-associated small molecules from chemically complex marine matrices and the subsequent validation of their biological effects beyond target-binding evidence. Conventional isolation-first strategies are time-consuming and may lead to the repeated purification of compounds with limited target relevance. Therefore, an integrated strategy linking target-guided screening, computational prioritization, compound isolation, cellular evaluation, and in vivo verification is needed.

The conventional natural product research paradigm of “chemical isolation first, followed by activity evaluation” is often inefficient when applied to complex matrices such as marine medicinal algae, which are characterized by wide polarity ranges, abundant isomers, and diverse degrees of polymerization. Under these conditions, isolation-first workflows may suffer from low efficiency, high off-target rates, and frequent false-positive selection [22]. To overcome these limitations, we established an integrated target-driven platform for the discovery and pharmacological validation of bioactive natural products [23,24]. Specifically, using CTSB as the target, affinity ultrafiltration LC-MS (UF-LC-MS) was employed for the high-throughput screening of active compounds, enabling the rapid identification of candidate ligands with potential binding affinity for CTSB [25,26]. Molecular docking and molecular dynamics simulations were then used to characterize possible binding modes, identify key amino acid residues, and evaluate complex stability, thereby reducing the likelihood of false-positive selection [27]. High-speed counter-current chromatography (HSCCC), combined with semi-preparative high-performance liquid chromatography (semi-preparative HPLC), was further applied for preparative-scale isolation, followed by structural characterization using LC-MS/MS and NMR [28,29,30]. The CTSB-associated activity of the isolated compounds was then evaluated using cellular assays, and a nude mouse subcutaneous xenograft model was used to assess the in vivo antitumor efficacy and preliminary safety of *S. polycystum*.

This study therefore established a stepwise workflow integrating high-throughput affinity screening, in silico validation, targeted isolation, cellular evaluation, and in vivo verification. By combining UF-LC-MS-based target screening, computational prioritization, preparative chromatography, structural identification, and pharmacological assessment, this strategy provides a practical approach for identifying CTSB-associated bioactive metabolites from chemically complex marine algal matrices. The findings support *S. polycystum* as a promising marine medicinal algal resource for the discovery of antitumor candidates with potential anti-NSCLC relevance. Further studies involving direct enzymatic kinetics, target-engagement assays, and genetic validation are warranted to clarify the causal role of CTSB in the observed antitumor effects.

## 2. Results

### 2.1. Affinity Ultrafiltration Screening and Matrix-Based Comprehensive Evaluation

#### 2.1.1. Affinity UF-LC-MS Screening Analysis

**Figure 1:**
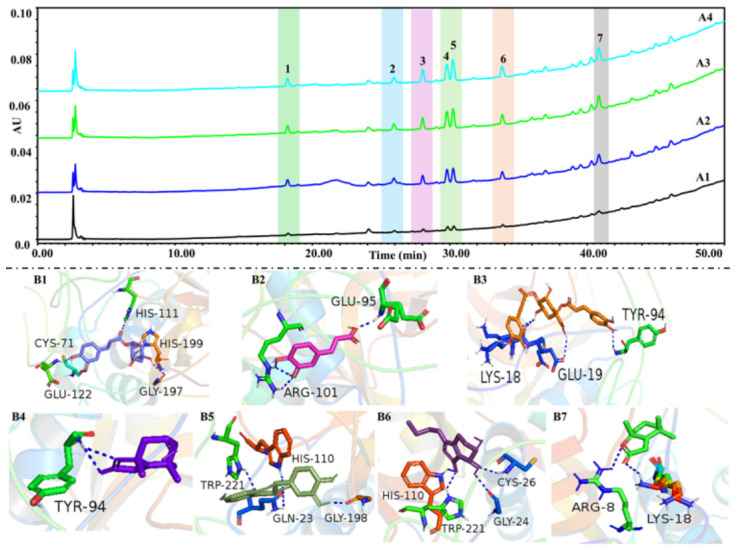
Chromatograms of CTSB-binding active compounds screened from *S. polycystum* by affinity UF-LC-MS and their molecular docking analysis with CTSB. (**A1**–**A4**) Chromatograms obtained in the control and at CTSB concentrations of 0.5, 1.0, and 2.5 U/mL, respectively. Peaks **1**–**7** correspond to chlorogenic acid, caffeic acid, cynarin, loliolide, taxifolin, senkyunolide H, and dihydroactinidiolide, respectively. (**B1**–**B7**) Three-dimensional docking models of these active compounds with CTSB, showing their interactions with key amino acid residues in the binding pocket.

Affinity UF-LC-MS screening identified seven potential CTSB-binding compounds from the *S. polycystum* extract, namely chlorogenic acid, caffeic acid, cynarin, loliolide, taxifolin, senkyunolide H, and dihydroactinidiolide (Figure 1). The binding degrees of these compounds increased markedly with increasing CTSB concentration, indicating a concentration-dependent enhancement of target-associated binding. At a CTSB concentration of 2.5 U/mL, all seven candidate compounds exhibited binding degrees above 70%, suggesting measurable and relatively strong interactions with CTSB (Table 1). These results indicate that the selected metabolites possess potential affinity for CTSB and warrant further computational and biological validation.

Recovery rate analysis was further performed to evaluate compound recovery after ultrafiltration and to assess the stability of compound–CTSB interactions during the UF process (Table 1). Among the tested compounds, caffeic acid exhibited the highest recovery rates across different CTSB concentrations, suggesting relatively stable binding and favorable elution behavior. Although most screened compounds showed high binding degrees, their recovery rates were generally below 90%, suggesting that their interactions with CTSB may be reversible and predominantly non-covalent rather than irreversible covalent binding. Overall, the affinity ultrafiltration results provide preliminary evidence that these seven metabolites have CTSB-binding potential and may serve as candidates for subsequent target-oriented prioritization and pharmacological evaluation.

#### 2.1.2. Matrix-Based Comprehensive Evaluation of Affinity UF-LC-MS Results

To further integrate the affinity UF-LC-MS results, the seven active compounds were comprehensively evaluated by jointly considering their binding degree (BD) and recovery rate (RR). A UF evaluation matrix was established to systematically assess the performance of each compound across different CTSB concentrations. Based on the experimental data presented in Table 1, separate matrices for BD and RR were constructed, and the overall binding performance of the candidate compounds was analyzed in a multidimensional manner as follows:

$$\mathrm{Binding}\;\mathrm{Degree}\;(\%)\;=\;\left[\begin{array}{c} \begin{array}{ccc} 65.65 & 71.71 & 77.25\\ 63.32 & 74.64 & 73.99\\ 70.22 & 82.06 & 84.94 \end{array}\\ \begin{array}{ccc} 73.49 & 79.21 & 77.64\\ 69.95 & 78.08 & 81.56\\ 70.34 & 81.78 & 82.67 \end{array}\\ \begin{array}{ccc} 76.63 & 83.56 & 85.61 \end{array} \end{array}\right],\;\mathrm{RR}\;(\%)\;=\;\left[\begin{array}{c} \begin{array}{ccc} 45.13 & 54.78 & 68.12\\ 61.03 & 88.26 & 86.07\\ 19.57 & 32.48 & 38.68 \end{array}\\ \begin{array}{ccc} 48.79 & 62.21 & 57.83\\ 25.53 & 35.00 & 41.61\\ 24.62 & 40.09 & 42.15 \end{array}\\ \begin{array}{ccc} 30.63 & 43.56 & 49.76 \end{array} \end{array}\right]$$

The comprehensive inhibition rates of seven compounds with different CTSB activities were calculated, with BDmax (85.61) and RRmax (88.26) as the maximum values of BD and RR, respectively, and the matrix for the comprehensive inhibition rate was then obtained by performing calculations and is as follows:

$$\mathrm{Comprehensive}\;\mathrm{inhibition}\;\mathrm{rate}\;=\;\left[\begin{array}{c} \begin{array}{ccc} 0.3921 & 0.3928 & 0.5262\\ 0.3864 & 0.6588 & 0.6368\\ 0.1374 & 0.2665 & 0.3285 \end{array}\\ \begin{array}{ccc} 0.3586 & 0.4928 & 0.4490\\ 0.1786 & 0.2733 & 0.3394\\ 0.1732 & 0.3279 & 0.3485 \end{array}\\ \begin{array}{ccc} 0.2347 & 0.3640 & 0.4260 \end{array} \end{array}\right]$$

The comprehensive inhibition rates of the seven active compounds with different CTSB activities were obtained from the matrix of the comprehensive inhibition rates, and the comprehensive score for each chemical component was calculated. Taking compound **1** as an example, the results at three enzyme concentrations are listed in the top row of the matrix. The calculated score was (0.2963 + 0.3928 + 0.5262)/3 = 0.4051, and the remaining six compounds were calculated in the same way. After calculation, the average comprehensive inhibition rates for each compound under different CTSB activities were 0.4051, 0.5607, 0.2442, 0.4334, 0.2637, 0.2832 and 0.3416 respectively, and the average comprehensive inhibition rate of CTSB followed the order 2 > 4 > 1 > 7 > 6 > 5 > 3, from largest to smallest. In addition, the obtained data can be used, along with other experimental data, to rank compound activity using matrix and grayscale analyses.

### 2.2. Computational Modeling of Active Compounds Binding to CTSB Target Proteins

#### 2.2.1. Docking Results

According to the UF-LC-MS experimental screening, the seven active compounds (chlorogenic acid, caffeic acid, cynarin, loliolide, taxifolin, senkyunolide H, and dihydroactinidiolide) were further verified by molecular docking technology, as shown in Figure 1(B1–B7). Seven active compounds dock to the 8b4t target protein (Figure S1). In molecular docking, the parameter binding energy can represent the binding capacity of receptors and ligands. As shown in Table 2, larger absolute values indicate stronger binding.

The docking analysis showed that all seven CTSB inhibitors formed stable complexes with the CTSB target protein, driven by hydrogen bonding, salt bridge, and other non-covalent interactions, with additional stabilization from hydrogen bonds to specific amino acid residues. Detailed interaction mapping showed that chlorogenic acid bound to CYS 71, HIS 111, HIS 199, GLU 122, and GLY 197; caffeic acid to GLU 95, ARG 101; cynarin to LYS 18, GLU 19, TYR 94; loliolide to TYR 94; taxifolin to GLN 23, HIS 110, GLY 198, and TRP 221; senkyunolide H to GLY 24, CYS 26, HIS 110, and TRP 221; and dihydroactinidiolide to ARG 8, LYS 18. The predicted binding free energies were −8.2, −6.5, −9.4, −6.3, −8.0, −7.3, and −6.4 kcal/mol for the seven compounds (CA-074Me was −10.2 kcal/mol), respectively, indicating favorable and spontaneous binding (Table 2).

Molecular docking provides an intuitive means of visualizing small molecule–protein interactions, and the current results confirm that the protein models used are of high quality. The identified CTSB are likely to constitute the key chemical basis for the activity of *S. polycystum*. Nevertheless, as docking relies on static stoichiometric models, it can only partially represent the in vivo behavior of ligands and is susceptible to false positives. Consequently, docking predictions must be corroborated through chromatographic analyses and in vitro bioassays. To refine these findings, conformations with low binding energies and high research relevance were subjected to molecular dynamics simulations. This approach offers deeper insight into the interaction mechanisms of major active compounds with critical protein targets implicated, thereby compensating for potential limitations inherent in docking and experimental assays.

#### 2.2.2. Dynamics Simulation Results

**Figure 2:**
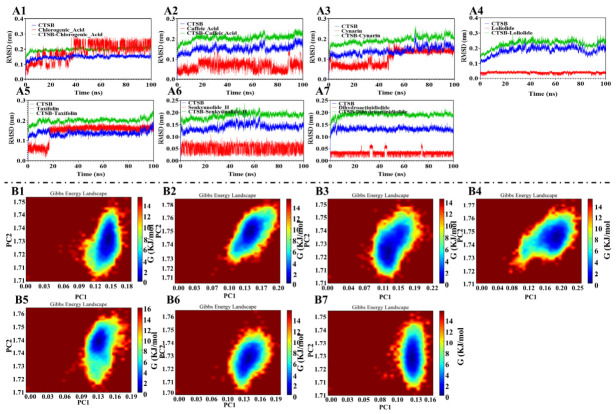
Molecular dynamics simulation-based RMSD analysis and Gibbs free energy landscapes of CTSB in complex with active compounds from *S. polycystum*. (**A1**–**A7**) RMSD plots of free CTSB and CTSB bound to the active compounds. (**B1**–**B7**) Gibbs free energy landscapes of the corresponding CTSB–ligand complexes generated from molecular dynamics trajectories. Compounds **1**–**7** correspond to chlorogenic acid, caffeic acid, cynarin, loliolide, taxifolin, senkyunolide H, and dihydroactinidiolide, respectively.

Through MD simulations, the dynamic stability of the CTSB–ligand complexes was further evaluated. As shown in Figure 2(A1–A7), the RMSD profiles of the seven complexes all exhibited a certain degree of conformational adjustment during the initial stage of the simulation and then gradually reached a plateau, indicating that all systems achieved a relatively stable state during the 100 ns simulation period. Among them, the caffeic acid and cynarin complexes showed relatively larger fluctuations at the early stage, suggesting more pronounced conformational adaptation of the protein upon ligand binding; however, their trajectories gradually stabilized in the later stage, indicating that the overall complexes still maintained favorable binding stability.

Free-energy landscape analysis further showed that the different ligand–CTSB complexes occupied one or more relatively stable low-energy conformational states during the simulations, suggesting that these ligands were able to adopt thermodynamically favorable conformations upon binding. Per-residue energy decomposition analysis (Figure 2(B1–B7) and Figure S2(b1–b7)) revealed that several key amino acid residues contributed substantially to ligand binding, indicating that these hotspot residues played important roles in maintaining complex stability. For caffeic acid and cynarin, their interactions with CTSB appeared to be more prominently driven by the combined contributions of van der Waals and electrostatic interactions.

Hydrogen-bond analysis (Figure S2(c1–c7)) showed that the number of protein–ligand hydrogen bonds fluctuated only slightly throughout the simulations, suggesting that relatively persistent intermolecular interactions were maintained after complex formation. Although the absolute number of hydrogen bonds varied among ligands, the overall trend indicated that most complexes preserved relatively stable binding modes during the entire simulation period. The radius of gyration (Rg) analysis (Figure S2(d1–d7)) showed that the Rg values of all complexes remained generally stable during the simulations, indicating that ligand binding did not cause obvious destabilization of the overall CTSB conformation. Notably, the complexes with caffeic acid and cynarin exhibited relatively smaller and more stable Rg values, suggesting that the protein maintained a comparatively compact conformational state in the presence of these two ligands. Secondary-structure evolution analysis (Figure S2(e1–e7)) demonstrated that CTSB underwent a certain degree of local conformational rearrangement upon ligand binding during the early stage of the simulation, whereas the overall secondary structure remained relatively stable during the subsequent trajectory, with no apparent structural collapse observed. These findings suggest that ligand binding did not markedly disrupt the structural integrity of the protein scaffold.

Consistent with the above MD analyses, the MM/GBSA results showed that the total binding free energies (ΔTOTAL) of all ligands toward CTSB were negative, indicating thermodynamically favorable binding (Table 3). Among them, cynarin exhibited the most favorable predicted binding free energy (ΔTOTAL = −28.25 kcal/mol), followed by taxifolin (ΔTOTAL = −26.68 kcal/mol) and caffeic acid (ΔTOTAL = −20.45 kcal/mol), whereas dihydroactinidiolide showed a relatively higher binding free energy (ΔTOTAL = −11.45 kcal/mol), indicating comparatively weaker binding stability to CTSB. For the ligands with stronger binding, the favorable interaction was mainly attributed to van der Waals and electrostatic contributions, although solvation energy partially offset these binding effects. In contrast, the weaker binding tendency of dihydroactinidiolide was associated with its relatively smaller van der Waals and electrostatic energy contributions.

Taken together, the RMSD, hydrogen bond, Rg, and MM/GBSA results suggest that several of the screened compounds, particularly caffeic acid, cynarin, and taxifolin, were able to form relatively stable complexes with CTSB. Overall, the binding of these ligands to CTSB appeared to be primarily driven by van der Waals interactions, together with the cooperative contributions of electrostatic interactions and hydrogen bonding. These computational findings were generally consistent with the preceding docking and experimental screening results, thereby providing support for subsequent structure–activity relationship analysis and CTSB-related mechanistic investigation.

### 2.3. Activity Analysis of High-Throughput Screening Through Gray Relational Analysis

**Figure 3:**
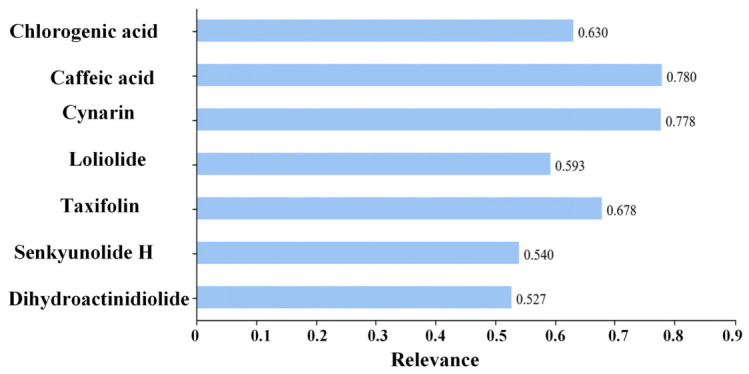
Correlation coefficients between the compounds and various biological activities, including UF, molecular docking, dynamic simulation.

To enable cross-comparison among datasets with different scales and dimensions, all experimental variables were normalized to a 0–1 range using min–max normalization. Based on the standardized values, an integrated score was calculated for each compound using predefined weights assigned to the UF-LC-MS, molecular docking, and MD simulation datasets. The normalized data and composite scores are summarized in Table 4. On this basis, GRA was performed to further evaluate the overall performance of the seven candidate compounds in a multidimensional manner (Figure 3).

Using the compound with the highest composite score, caffeic acid, as the reference sequence, the gray relational degrees (GRGs) of the remaining compounds were calculated relative to this profile. As shown in Table 4, the GRGs of the seven candidate compounds ranged from 0.527 to 0.780, and the overall ranking was as follows: caffeic acid (**2**) > cynarin (**3**) > taxifolin (**5**) > chlorogenic acid (**1**) > loliolide (**4**) > senkyunolide H (**6**) > dihydroactinidiolide (**7**). Among them, caffeic acid exhibited the highest GRG, indicating the closest overall similarity to the optimal reference profile across the integrated evaluation indices. Cynarin ranked second and showed consistently favorable performance, particularly in the docking and MD analyses. Taxifolin also displayed a relatively high GRG, suggesting good integrated activity despite its comparatively lower UF-LC-MS-derived score. In contrast, dihydroactinidiolide showed the lowest GRG, indicating the weakest overall performance among the seven compounds evaluated.

Taken together, the GRA results provided a multidimensional prioritization of CTSB-targeting constituents from *S. polycystum* by integrating experimental screening and computational validation. Rather than relying on a single index, this approach allowed for a more systematic comparison of compounds across multiple evaluation dimensions and supported the prioritization of caffeic acid, cynarin, and taxifolin for subsequent preparative isolation and biological validation. These findings further indicate that the combined use of affinity-based screening, computational modeling, and GRA is a practical strategy for the discovery and ranking of bioactive CTSB-related natural products from complex marine medicinal matrices.

### 2.4. Separation of Active Compounds from S. polycystum Using Complex Chromatography

Based on the combined results of affinity UF-LC-MS, molecular docking, and MD dynamics, seven CTSB-targeted compounds were selected for preparative separation. Considering their structural diversity (including phenolic acids, flavonoids, and lactones), a comprehensive chromatographic workflow was employed to achieve high-yield and high-purity isolation.

#### 2.4.1. Optimization of Separation Parameters for CCC

Guided by in vitro activity screening, seven active compounds were chosen for targeted isolation using a combined chromatographic approach. Owing to the wide variation in partition coefficients (K-values) among these compounds, eleven ternary and quaternary biphasic solvent systems with different polarities were systematically assessed (Table 5). Several systems showed clear drawbacks. After comparing and analyzing eleven solvent systems, the solvent system consisting of ethyl acetate:methanol:*n*-butanol:water (5:2:2.5:9, *v*/*v*/*v*/*v*) was identified as the most suitable for separation. This system demonstrated a well-balanced separation efficiency for all compounds. Compared to other solvent systems, it effectively separated compounds such as chlorogenic acid, cynarin, taxifolin, and senkyunolide H, while also maintaining a moderate yet efficient separation of dihydroactinidiolide. This avoids the extreme separation issues observed with some compounds in other systems. Notably, when separating multiple compounds, it offers a balanced approach, not favoring any single compound, yet achieving optimal separation performance. Moreover, the proportion of ethyl acetate, methanol, n-butanol, and water in the system provides excellent solubility and fluidity, further enhancing separation efficiency. Therefore, this system emerges as the ideal choice, particularly for separation studies involving a wide range of compounds, offering strong versatility and practical application value. In contrast, while other solvent systems may excel in separating specific compounds, they often encounter issues with imbalanced separation or suboptimal performance for certain compounds. As a result, with its overall superior performance, the ethyl acetate:methanol:*n*-butanol:water (5:2:2.5:9, *v*/*v*/*v*/*v*) system stands out as the optimal choice for multi-target separation studies.

#### 2.4.2. Separation by CCC

**Figure 4:**
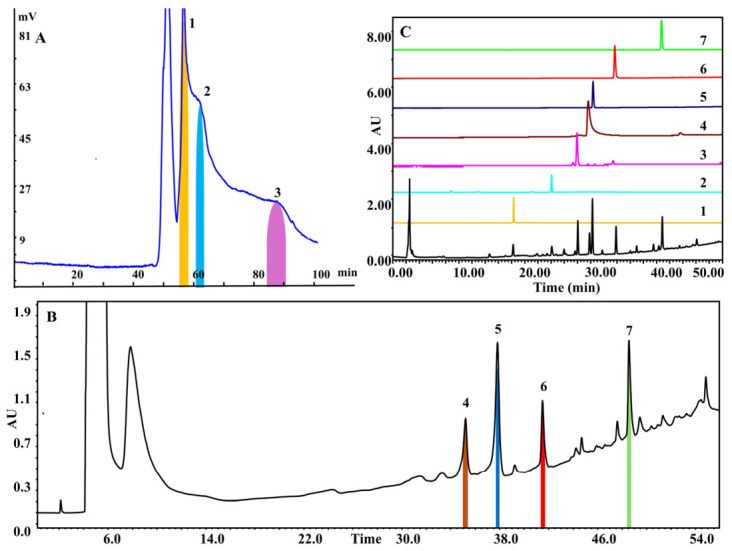
Isolation and purification of active compounds from *S. polycystum* using complex chromatography techniques. (**A**) CCC chromatogram of the *S. polycystum* extract. (**B**) Semi-preparative HPLC chromatogram of the *S. polycystum* extract. (**C**) HPLC showing the purity of the isolated compounds. Compounds **1**–**7** correspond to chlorogenic acid, caffeic acid, cynarin, loliolide, taxifolin, senkyunolide H, and dihydroactinidiolide, respectively.

The CCC separation of *S. polycystum* was carried out with a flow rate of 6.0 mL/min, using a solvent system of ethyl acetate:methanol:*n*-butanol:water, 800 rpm, and detection at 254 nm (Figure 4A). Three compounds—chlorogenic acid, caffeic acid, and cynarin—were isolated with purities of 95.15, 92.31, and 90.16%, respectively. CCC separations were carried out for a total duration of 100 min, ensuring stable retention of the stationary phase.

#### 2.4.3. Semi-Preparative HPLC Separation of Active Compounds

Owing to the limitations of CCC, some of the compounds isolated in this study displayed lower purity. Therefore, semi-preparative HPLC was employed for further separation. The results showed that methanol was the most effective eluent for separating individual compounds from the *S. polycystum* extract. Furthermore, the findings revealed that higher flow rates resulted in shorter retention times. However, this also led to reduced separation efficiency over time, and the increased pressure within the column could shorten its operational lifespan. The target compounds obtained at the 3.0 mL/min flow rate was selected. The semi-preparative HPLC profile of *S. polycystum* is shown in Figure 4B. Four compounds—loliolide, taxifolin, senkyunolide H, and dihydroactinidiolide—were successfully purified in a brief period, yielding compounds of high purity. The HPLC-determined purities were 90.03, 94.76, 95.39 and 93.17%, respectively. These results demonstrate that semi-preparative HPLC effectively complements CCC by achieving the high-purity isolation of key compounds, supporting downstream biological evaluation and potential scale-up.

### 2.5. Separation Results and Individual Compounds Analysis

Seven individually separated and identified compounds from *S. polycystum* were analyzed using HPLC with the area normalization method. The purity of each compound exceeded 90%, with the following purities for fractions 1 to 7: 95.15, 92.31, 90.16, 90.03, 94.76, 95.39, and 93.17%. The LC chromatograms of both the extract and the chromatography fractions are displayed in Figure 4C. Table 6 compares the MS data, the LC retention times, and NMR of the target individual compounds, which include chlorogenic acid, caffeic acid, cynarin, loliolide, taxifolin, senkyunolide H, and dihydroactinidiolide [31,32,33,34,35,36,37].

Compound **1**: Chlorogenic acid, C_16_H_18_O_9_, 353.0885 [M-H]^−^. The 1H-NMR (MeOD, 500 MHz) δ: 2.16–2.24 (2H, m, H-3), 4.15–4.18 (1H, m, H-4), 3.71–3.74 (1H, m, H-5), 5.31–5.35 (2H, m, H-6), 2.03–2.10 (1H, m, H-7), 6.24/6.28 (1H, t, H-9), 7.54/7.57 (1H, t, H-10), 7.04/7.05 (1H, t, H-12), 6.94–6.96 (1H, m, H-15), 6.77/6.78 (1H, t, H-16). 13C-NMR (MeOD, 126 MHz) δ: 177.04 (C-1), 76.15 (C-2), 38.79 (C-3), 72.01 (C-4), 71.30 (C-5), 38.25 (C-6), 73.48 (C-7), 168.69 (C-8), 115.29 (C-9), 146.85 (C-10), 127.84 (C-11), 115.23 (C-12), 146.85 (C-13), 149.62 (C-14), 116.51 (C-15), 123.02 (C-16).

Compound **2**: Caffeic acid, C_9_H_8_O_4_, 181.0455 [M+H]^+^. The 1H-NMR (MeOD, 500 MHz) δ: 7.55/7.51 (1H, d, H-7), 7.03 (1H, d, H-2), 6.94/6.92 (1H, m, H-6), 6.78/6.77 (1H, d, H-5), 6.23/6.20 (1H, d, H-8). 13C-NMR (MeOD, 126 MHz) δ: 127.84 (C-1), 115.12 (C-2), 147.09 (C-3), 149.51 (C-4), 115.56 (C-5), 122.89 (C-6), 146.86 (C-7), 116.53 (C-8), 171.06 (C-9).

Compound **3**: Cynarin, C_25_H_24_O_12_, 517.1353 [M+H]^+^. The 1H-NMR (500 MHz, MeOD) δ 7.58 (2H, d, H-7′, 7″), 7.06 (2H, t, H-2′, 2″), 6.96 (2H, dt, H-6′, 6″), 6.78 (2H, dd, H-5, 5′), 6.28 (2H, t, H-8′, 8″), 5.38 (1H, m, H-5), 4.29 (1H, dd, H-3), 3.78 (1H, dd, H-4), 2.56 (1H, dd, H-2a), 2.44 (2H, m, H-2b, 6b), 2.03 (1H, m, H-6a). 13C-NMR (126 MHz, MeOD) δ 80.87 (C-1), 35.64 (C-2), 69.38 (C-3), 72.76 (C-4), 71.59 (C-5), 36.88 (C-6), 174.75 (C-7), 127.84 (C-1′, 1″), 115.25 (C-2′), 115.19 (C-2″), 146.87 (C-3′), 146.86 (C-3″), 149.72 (C-4′), 149.67 (C-4″), 116.54 (C-5″), 116.51 (C-5″), 123.15 (C-6′), 123.07 (C-6″), 147.59 (C-7″), 147.36 (C-7′), 115.27 (C-8′), 115.15 (C-8″), 168.65 (C-9′), 168.04 (C-9″).

Compound **4**: Loliolide, C_11_H_16_O_3_, 197.1169 [M+H]^+^. The 1H-NMR (500 MHz, DMSO) δ 5.78 (1H, s, H-2), 4.98 (1H, d, H-6), 4.08 (1H, m, H-OH), 2.30/2.27 (1H, m, H-7a), 1.64/1.62 (1H, m, H-7b),1.88/1.85 (1H, m, H-5a), 1.43/1.40 (1H, m, H-5b), 1.67 (3H, s, H-11), 1.38 (3H, s, H-10), 1.19 (3H, s, H-9). 13C-NMR (126 MHz, DMSO) δ 171.05 (C-1), 112.11 (C-2), 183.07 (C-3), 35.67 (C-4), 46.62 (C-5), 64.85 (C-6), 45.27 (C-7), 86.49 (C-8) 30.43 (C-9), 26.21 (C-10), 26.83 (C-11).

Compound **5**: Taxifolin, C_15_H_12_O_7_, 303.0511 [M-H]^−^. The 1H-NMR (500 MHz, MeOD) δ 6.95 (1H, d, H-15), 6.82/6.84 (1H, dd, H-11), 6.78/6.79 (1H, d, H-12), 5.91 (1H, d, H-6), 5.87 (1H, d, H-4), 4.91/4.89 (1H, overlap, H-8), 4.48/4.50 (1H, d, H-9). 13C-NMR (126 MHz, MeOD) δ 198.49 (C-1), 101.89 (C-2), 164.57 (C-3), 97.33 (C-4), 168.73 (C-5), 96.30 (C-6), 165.37 (C-7), 85.18 (C-8), 73.72 (C-9), 129.90 (C-10), 120.94 (C-11), 116.11 (C-12), 147.20 (C-13), 146.37 (C-14), 115.91 (C-15).

Compound **6**: Senkyunolide H, C_12_H_16_O_4_, 223.0971 [M-H]^−^. The 1H-NMR (500 MHz, DMSO) δ 5.44 (1H, t, H-13), 4.99 (1H, d, H-11), 4.69 (1H, d, H-12), 4.20 (1H, m, H-9), 3.59 (1H, m, H-8), 2.57/2.39 (2H, m, H2–6), 2.25 (2H, m, H2–14), 1.80/1.65 (2H, m, H2–7), 1.45 (2H, dt, H2–15), 0.90 (3H, t, H-16). 13C-NMR (126 MHz, DMSO) δ 167.73 (C-2), 153.34 (C-5), 147.76 (C-4), 127.01 (C-3), 112.49 (C-13), 68.58 (C-8), 61.29 (C-9), 27.58 (C-14), 24.37 (C-7), 21.74 (C-15), 19.82 (C-6), 13.66 (C-16).

Compound **7**: Dihydroactinidiolide, C_11_H_16_O_2_, 179.1071 [M-H]^−^. The 1H-NMR (DMSO, 500 MHz) δ: 5.76 (1H, s, H-2), 1.36 (2H, m, H-5), 1.68 (2H, m, H-6), 1.73/2.17 (2H, m, H-7), 1.50 (3H, s, H-9), 1.23 (3H, s, H-10), 1.19 (3H, s, H-11). 13C-NMR (DMSO, 126 MHz) δ: 170.97 (C-1), 111.78 (C-2), 182.63 (C-3), 36.17 (C-4), 39.75 (C-5), 19.06 (C-6), 41.05 (C-7), 86.75 (C-8), 29.54 (C-9), 24.09 (C-10), 23.77 (C-11).

### 2.6. In Vitro Cellular Biological Evaluation

#### 2.6.1. Evaluation of the In Vitro Biological Effects of Active Compounds

Previously published results demonstrated that different active monomeric compounds exhibited varying degrees of inhibitory effects on NCI-H1975 cells in vitro. Their IC_50_ values were 33.54, 6.29, 27.93, 27.20, 26.11, 26.88, and 29.67 μg/mL for chlorogenic acid, caffeic acid, cynarin, loliolide, taxifolin, senkyunolide H, and dihydroactinidiolide, respectively [38]. Among these compounds, caffeic acid showed the strongest inhibitory activity, suggesting marked differences in the anti-NSCLC effects of the tested monomers. These results indicate that all of these active compounds are capable of interfering with the growth of NCI-H1975 cells in vitro and provide a basis for subsequent target-based activity screening and mechanistic investigation.

#### 2.6.2. Inhibitory Effects of Active Compounds on Intracellular CTSB Activity

The results showed that active compounds inhibited intracellular CTSB activity in NCI-H1975 cells to varying extents, with an overall trend of enhanced inhibition as the treatment concentration increased, indicating a relatively clear concentration-dependent inhibitory effect on CTSB. Among these compounds, caffeic acid, chlorogenic acid, and cynarin exhibited relatively strong inhibitory activity against CTSB, followed by taxifolin, whereas loliolide, senkyunolide H, and dihydroactinidiolide showed comparatively weaker effects. Overall, phenolic acid-type compounds displayed stronger inhibitory effects than some lactone-type constituents, suggesting that they may represent more promising CTSB-targeting candidates within this active compound library (Table 7).

In this assay, equal amounts of total protein were used to construct the reaction system, and Z-Arg-Arg-AMC was employed as a fluorogenic substrate to measure CTSB-catalyzed hydrolysis, thereby minimizing interference caused by differences in protein loading. Accordingly, the reduced fluorescence signals in the treated groups are more likely to reflect the suppression of CTSB enzymatic activity itself rather than fluctuations in total protein content. In addition, Z-Arg-Arg-AMC has been widely used as a substrate for CTSB activity assays in related studies, supporting the methodological reliability and credibility of the present results. These active compounds, especially the phenolic acid-type constituents with stronger inhibitory activity, may therefore serve as potential CTSB-targeting molecules. The present findings also provide an experimental basis for further investigation into whether these compounds exert anti-NSCLC effects through the regulation of CTSB and related cell death pathways.

#### 2.6.3. Immunofluorescence Analysis of Intracellular CTSB-Related Signals

**Figure 5:**
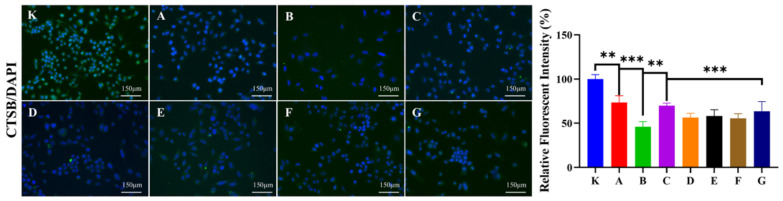
Immunofluorescence analysis of intracellular CTSB-related fluorescence signals in NCI-H1975 cells after treatment with different active monomeric compounds. (**A**) chlorogenic acid, 33.54 μg/mL; (**B**) caffeic acid, 6.291 μg/mL; (**C**) cynarin, 27.93 μg/mL; (**D**) loliolide, 27.20 μg/mL; (**E**) taxifolin, 26.11 μg/mL; (**F**) senkyunolide H, 26.88 μg/mL; (**G**) dihydroactinidiolide, 29.67 μg/mL; K, control group. Green fluorescence indicates CTSB-related signals, and blue fluorescence indicates DAPI-stained nuclei. Images were acquired under the same magnification and identical imaging parameters. Representative fields with comparable cell density are shown. Scale bar = 150 μm. Quantitative fluorescence intensity data are presented as mean ± SD. Statistical analysis was performed using GraphPad Prism 10.2.0 (San Diego, CA, USA). One-way ANOVA followed by Tukey’s post hoc test was used. ** *p* < 0.01 and *** *p* < 0.001 versus the control group.

To further evaluate the effects of the active compounds on CTSB-associated intracellular fluorescence in NCI-H1975 cells, immunofluorescence staining followed by ImageJ 1.53 based quantification was performed (Figure 5 and Figure S3). CTSB immuno-fluorescence was predominantly observed in the cytoplasm, showing punctate to partially diffuse distribution patterns that were broadly consistent with the known endolysosomal/lysosomal localization of CTSB. Compared with the control group, the green fluorescence intensity of CTSB was reduced in all treatment groups, indicating that the active monomers generally decreased the intracellular CTSB-associated immunofluorescence signal. Quantitative analysis showed that caffeic acid produced the most pronounced reduction in CTSB fluorescence intensity, followed by chlorogenic acid and cynarin. Loliolide, taxifolin, and senkyunolide H caused moderate decreases, whereas dihydroactinidiolide showed the weakest effect, although its fluorescence intensity remained significantly lower than that of the control group. Overall, CTSB-associated fluorescence was reduced to different extents across all treatment groups, revealing a clear gradient in intracellular CTSB-related signal intensity.

Notably, this trend was generally consistent with the preceding CTSB enzymatic assay, in which several active monomers also showed inhibitory effects on CTSB activity. In particular, the phenolic acid-type compounds, especially caffeic acid, chlorogenic acid, and cynarin, exhibited relatively stronger effects than several lactone-type compounds in both assays. Taken together, these findings suggest that the active compounds may modulate CTSB-related cellular behavior at both the enzymatic and immunofluorescence levels, thereby supporting their prioritization for further CTSB-related mechanistic investigation in NSCLC cells.

### 2.7. In Vivo Validation

#### 2.7.1. Effects of *S. polycystum* on NCI-H1975 Xenograft Tumor Growth in Nude Mice

**Figure 6:**
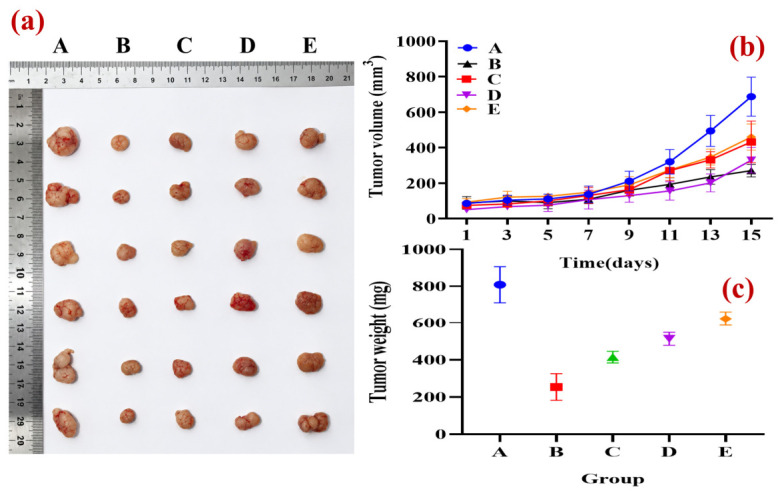
Inhibitory effects of *S. polycystum* on tumor growth in NCI-H1975 xenograft nude mice. (**a**) Representative images of excised tumors from each group; (**b**) tumor growth curves; (**c**) comparison of final tumor weights. A–E, model group, positive control group (paclitaxel, 10 mg/kg), *S. polycystum* high-dose group (1.5 g/kg), *S. polycystum* middle-dose group (1.0 g/kg), and *S. polycystum* low-dose group (0.5 g/kg), respectively. Data are presented as mean ± SD (*n* = 6). Tumor growth curves were analyzed using two-way ANOVA followed by multiple comparisons, and final tumor weights were analyzed using one-way ANOVA followed by Tukey’s post hoc test. Exact *p* values are provided in the Results section. *p* < 0.05 and *p* < 0.01 indicate significant differences compared with the model group.

As shown in Figure 6, compared with the model group, the positive control group (paclitaxel, 10 mg/kg) and the *S. polycystum*-treated groups suppressed the growth of NCI-H1975 xenograft tumors in nude mice to varying degrees, as evidenced by reduced external tumor burden, smaller excised tumor masses, and decreased final tumor weights. Statistical analysis of final tumor weights was performed using one-way ANOVA followed by Tukey’s post hoc test. Compared with the model group, final tumor weight was significantly reduced in the positive control group (*p* = 0.005), as well as in the high-, middle-, and low-dose *S. polycystum* groups (*p* = 0.008, *p* = 0.016, and *p* = 0.037, respectively).

The tumor growth curves further showed that tumor volume in the model group increased rapidly throughout the experimental period, whereas tumor growth was markedly slowed in all treatment groups. Tumor volume data were analyzed using two-way ANOVA followed by multiple comparisons in GraphPad Prism 10.2.0 (San Diego, CA, USA). Compared with the model group, tumor growth was significantly inhibited in the positive control group (*p* = 0.003) and in the high-, middle-, and low-dose *S. polycystum* groups (*p* = 0.006, *p* = 0.014, and *p* = 0.041, respectively). Among all treatment groups, the positive control group exhibited the strongest antitumor effect. Within the *S. polycystum*-treated groups, the high-dose group (1.5 g/kg) showed the greatest inhibitory effect, followed by the middle-dose group (1.0 g/kg), whereas the low-dose group (0.5 g/kg) showed a relatively weaker effect, indicating a dose-dependent inhibitory trend.

The final tumor weight data further confirmed these findings. Compared with the model group, the tumor inhibition rate was 68.65% in the positive control group, while the inhibition rates in the high-, middle-, and low-dose *S. polycystum* groups were 48.78%, 36.58%, and 22.86%, respectively. Taken together, the tumor appearance, dynamic tumor volume changes, final tumor weight, and tumor inhibition rate results demonstrate that *S. polycystum* effectively inhibited the in vivo growth of NCI-H1975 xenograft tumors, with the high-dose group showing the most pronounced effect among the *S. polycystum*-treated groups. These findings provide an experimental basis for subsequent pathological and mechanistic investigations.

#### 2.7.2. Effects of *S. polycystum* on Tumor Histopathology and the Safety of Major Organs

As shown in Figure S4, H&E staining of liver and kidney tissues from all groups revealed relatively clear histological structures. In the normal control group, hepatic cords were regularly arranged, hepatocytes were morphologically intact, and the glomerular and renal tubular structures were clearly preserved. Similarly, no obvious severe histopathological damage was observed in the liver or kidney tissues of the model group, indicating that the tumor-bearing state itself did not cause significant histological abnormalities in these organs under the present experimental conditions. Compared with the model group, the positive control group and the high-, middle-, and low-dose *S. polycystum* groups all exhibited generally preserved liver and kidney morphology, without obvious extensive necrosis, marked inflammatory infiltration, severe disruption of hepatic cord architecture, glomerular atrophy, or tubular dilation. These findings suggest that *S. polycystum* did not induce evident hepatorenal histopathological toxicity under the present dosing conditions and thus showed a favorable preliminary in vivo safety profile.

**Figure 7:**
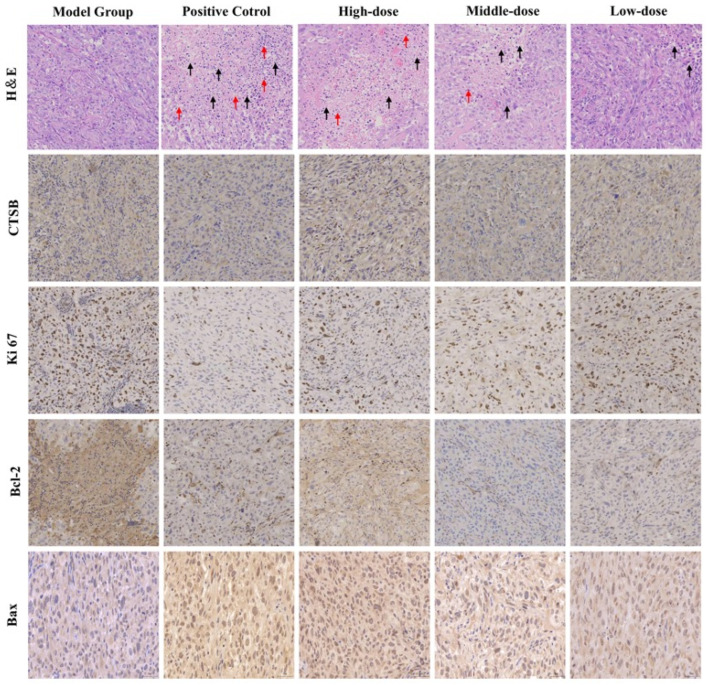
Effects of *S. polycystum* on tumor histopathology and the expression of CTSB, Ki-67, Bcl-2, and Bax in the model group, positive control group, high-dose group, middle-dose group, and low-dose group. Black arrows indicate tumor cell necrosis, and red arrows indicate inflammatory cell infiltration. Bright light and fluorescence microscopy.

H&E staining of tumor tissues showed that tumor cells in the model group were densely arranged and abundant (Figure 7), with hyperchromatic nuclei, marked pleomorphism, and evident atypia, but without obvious inflammatory cell infiltration, indicating an actively growing tumor state. In contrast, both the positive control group and the *S. polycystum*-treated groups exhibited varying degrees of histopathological injury, mainly characterized by loosened tumor cell arrangement, focal cell necrosis, inflammatory cell infiltration, and reduced nuclear atypia. These changes were most pronounced in the positive control group. In the *S. polycystum* groups, the high-dose group showed more evident tumor cell necrosis and mild inflammatory infiltration, the middle-dose group displayed moderate necrotic and inflammatory changes, whereas the low-dose group showed relatively limited histological alterations. Overall, *S. polycystum* induced degenerative and necrotic changes in tumor tissues to different extents, with a certain dose-dependent trend. These results are consistent with the observed changes in tumor volume, tumor weight, and tumor inhibition rate, further indicating that *S. polycystum* effectively suppresses tumor growth in vivo without causing apparent liver or kidney toxicity.

#### 2.7.3. Regulatory Effects of *S. polycystum* on Tumor Cell Proliferation, Apoptosis, and CTSB Expression

As shown in Figure 7, immunohistochemical staining revealed marked differences in the expression levels of CTSB, Ki-67, Bcl-2, and Bax among the different treatment groups. In the model group, strong positive staining for CTSB, Ki-67, and Bcl-2, together with relatively weak Bax staining, indicated high proliferative activity and a pronounced anti-apoptotic phenotype in tumor tissues (Figure 7).

Compared with the model group, overall CTSB staining was reduced in the positive control group as well as in all *S. polycystum*-treated groups, suggesting that treatment suppressed CTSB expression in tumor tissues. Combined with the previous in vitro enzymatic assays and the known roles of CTSB in lysosomal function, tumor invasion, and cell death regulation, these findings suggest that *S. polycystum* may interfere with malignant tumor-associated processes by downregulating CTSB expression (Figure 7).

Ki-67 staining showed abundant positive cells in the model group, whereas the positive control group and the *S. polycystum*-treated groups exhibited reduced Ki-67 staining, indicating that all treatments suppressed tumor cell proliferation to varying degrees. The decrease in Ki-67 expression was consistent with the previously observed reductions in tumor growth rate and final tumor weight, supporting an in vivo anti-proliferative effect of *S. polycystum* (Figure 7).

The Bcl-2 and Bax staining patterns further reflected changes in the apoptotic status of tumor cells. Strong Bcl-2 expression and weak Bax expression in the model group indicated a pronounced anti-apoptotic phenotype. In contrast, the positive control group and the *S. polycystum*-treated groups showed an overall decrease in Bcl-2 staining and an increase in Bax staining, suggesting an enhanced apoptotic tendency after treatment. These results imply that *S. polycystum* may promote tumor cell apoptosis by downregulating the anti-apoptotic protein Bcl-2 and upregulating the pro-apoptotic protein Bax, thereby modulating the mitochondrial apoptosis pathway (Figure 7).

Quantitative IHC analysis further confirmed these observations. Ki-67 positivity was highest in the model group (50.27%) and decreased to 13.62%, 20.70%, 26.00%, and 41.40% in the positive control, high-, middle-, and low-dose *S. polycystum* groups, respectively, indicating that *S. polycystum* inhibited tumor cell proliferation with an overall dose-dependent trend. CTSB expression was also highest in the model group (0.0959) and decreased to 0.0599, 0.0784, 0.0805, and 0.0811 in the positive control, high-, middle-, and low-dose groups, respectively, indicating that all treatments downregulated CTSB expression.

Quantitative analysis of Bcl-2 and Bax further showed that Bcl-2 expression was highest in the model group (0.1446), whereas Bax expression was lowest (0.0032). After treatment with the positive control or *S. polycystum*, Bcl-2 expression markedly decreased, whereas Bax expression significantly increased. In the *S. polycystum* low-, middle-, and high-dose groups, Bax expression increased to 0.0055, 0.0078, and 0.0104, respectively, showing an increasing trend with dose escalation. Overall, *S. polycystum* downregulated Ki-67, CTSB, and Bcl-2 expression while upregulating Bax expression, suggesting that it may exert its in vivo anti-NSCLC activity by suppressing tumor cell proliferation, reducing CTSB expression, and promoting apoptosis through modulation of the Bcl-2/Bax balance (Table 8).

#### 2.7.4. Effects of *S. polycystum* on Apoptosis and Ultrastructural Changes in Tumor Tissues

**Figure 8:**
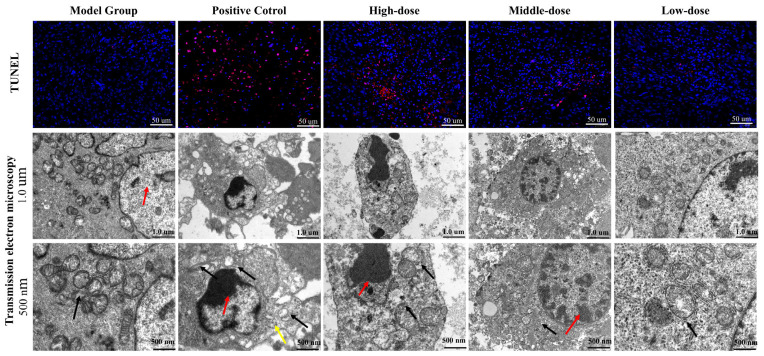
Effects of *S. polycystum* on apoptosis by TUNEL staining of tumor tissues (50 μm) and ultrastructural changes analyzed by transmission electron microscopy (1.0 μm; 500 nm) in the model, positive control, high-dose, middle-dose, and low-dose groups. Black arrows indicate mitochondrial damage such as swelling and cristae disruption/loss; red arrows indicate nuclear deformation or chromatin condensation; yellow arrows indicate vacuolization. Bright-field and transmission electron microscopy.

As shown in Figure 8, TUNEL staining revealed marked differences in the apoptotic levels of tumor cells among the different treatment groups. In the model group, only a few scattered TUNEL-positive signals were observed, indicating a low level of spontaneous apoptosis in tumor tissues. In contrast, the positive control group showed a marked increase in red fluorescent signals, suggesting significantly enhanced apoptosis. After treatment with *S. polycystum*, the number of TUNEL-positive cells increased in all dose groups compared with the model group, with the high-dose group showing the most pronounced increase, followed by the middle-dose group, whereas the low-dose group exhibited a relatively weaker effect. Overall, a dose-dependent trend was observed. These findings indicate that *S. polycystum* promotes tumor cell apoptosis, consistent with the previously observed upregulation of Bax and downregulation of Bcl-2.

Transmission electron microscopy further revealed the ultrastructural alterations of tumor cells in different treatment groups (Figure 8). In the model group, tumor cells retained relatively intact ultrastructure, with clear nuclear contours, orderly distribution of organelles, and only mild organelle alterations, indicating an actively growing tumor state. In contrast, the positive control group displayed pronounced ultrastructural damage, including nuclear pyknosis, chromatin margination, mitochondrial swelling or cristae disruption, organelle vacuolization, and partial membrane destruction, suggesting that treatment induced marked apoptotic and degenerative changes in tumor cells (Figure 8). Similar ultrastructural abnormalities were also observed in the *S. polycystum*-treated groups to varying extents. In the high-dose group, tumor cell damage was the most severe, characterized by nuclear pyknosis, chromatin condensation, abnormal mitochondrial morphology, cytoplasmic vacuolization, and partial alterations in lysosomal or membranous structures. The middle-dose group also exhibited evident organelle damage and nuclear abnormalities, although to a lesser extent than the high-dose group. The low-dose group showed mild mitochondrial and cytoplasmic alterations, with overall less severe damage.

Taken together, the TUNEL and TEM results demonstrate that *S. polycystum* significantly promotes tumor cell apoptosis and induces multiple ultrastructural changes, including nuclear pyknosis, chromatin condensation, mitochondrial injury, and cytoplasmic vacuolization, with the strongest effects observed in the high-dose group. These findings are consistent with the antitumor efficacy, H&E staining, and IHC results, and further suggest that *S. polycystum* may exert its in vivo anti-NSCLC activity by regulating the balance between proliferation and apoptosis while disrupting intracellular organelle homeostasis.

## 3. Discussion

Marine algae have attracted increasing attention as valuable sources of bioactive metabolites for anticancer drug discovery. Previous studies have shown that seaweed-derived polysaccharides, polyphenols, carotenoids, terpenoids, and sterols exert antitumor effects by inhibiting cancer cell proliferation, inducing apoptosis, regulating cell-cycle progression, suppressing angiogenesis, and modulating tumor-related signaling pathways [39]. Among brown algae, *Sargassum* species have been extensively investigated, particularly for fucoidan and other sulfated polysaccharides. Fucoidan isolated from *S. polycystum* has been reported to inhibit the proliferation of breast cancer and leukemia cells and induce mitochondria-mediated apoptosis [40]. In contrast to these studies, which mainly focused on polysaccharide fractions or crude extracts, the present study emphasized the CTSB-guided identification of small-molecule metabolites from *S. polycystum* and evaluated their CTSB-associated biological effects [21,41]. Thus, this work extends current knowledge of the antitumor material basis of *S. polycystum* from polysaccharide-dominated research to target-oriented small-molecule discovery.

CTSB was selected as the screening target because of its established involvement in tumor progression. Aberrant CTSB expression, secretion, or relocalization has been associated with extracellular matrix degradation, tumor invasion, metastasis, angiogenesis, and cell death regulation [16,42]. In lung cancer, elevated CTSB expression has also been linked to malignant progression and poor clinicopathological features. Consistent with these reports, *S. polycystum* extract reduced CTSB expression in xenograft tumor tissues. This finding indicates that the antitumor effect observed in the present study was accompanied by CTSB-related modulation. However, CTSB plays context-dependent roles in different tumor types and cellular processes; therefore, the present findings should be interpreted as evidence of CTSB-associated antitumor effects rather than definitive proof that CTSB is the sole or primary therapeutic target.

Affinity UF-LC-MS has been increasingly used to screen potential ligands from complex natural product matrices, as it allows target-binding compounds to be enriched before chromatographic and mass spectrometric identification [43,44]. Compared with conventional isolation-first strategies, this approach improves screening efficiency and enhances target relevance. In this study, affinity UF-LC-MS enabled the rapid identification of seven potential CTSB-binding metabolites from *S. polycystum*, and the concentration-dependent increase in binding degree further supported their possible interaction with CTSB. However, UF-LC-MS mainly reflects affinity enrichment and cannot independently confirm functional inhibition. Therefore, the candidate compounds were further evaluated by molecular docking, molecular dynamics simulation, chromatographic isolation, and biological assays.

The computational results supported the prioritization of the screened metabolites. Molecular docking suggested that the seven compounds could interact with CTSB through hydrogen bonding, electrostatic interactions, salt bridges, and other non-covalent contacts. Molecular dynamics simulations further indicated that caffeic acid, cynarin, and taxifolin may form relatively stable CTSB–ligand complexes, as reflected by RMSD, hydrogen bond, Rg, secondary structure, and MM/GBSA analyses. These results were broadly consistent with the UF-LC-MS and GRA findings, indicating that multidimensional screening is more reliable than a single evaluation index. Nevertheless, docking and molecular dynamics simulations remain predictive tools and require further confirmation through direct biochemical, enzymatic, and target-engagement assays.

The combined use of CCC and semi-preparative HPLC enabled the targeted isolation of seven structurally diverse metabolites from *S. polycystum*, including phenolic acids, flavonoids, and lactone-type constituents, all with purities above 90%. Although these compounds are known natural products, their CTSB-oriented screening, preparative recovery, and anti-NSCLC-related evaluation from *S. polycystum* have not been systematically integrated in previous studies. Therefore, the novelty of this work lies not in the discovery of new chemical structures, but in the establishment of a target-guided discovery and validation framework for identifying CTSB-associated antitumor metabolites from a chemically complex marine algal matrix.

The cellular assays showed that several isolated metabolites reduced CTSB-associated enzymatic activity and intracellular CTSB-related fluorescence signals in NCI-H1975 cells. These results were accompanied by reduced cell viability in the CCK-8 assay. Because CCK-8 mainly reflects cellular metabolic activity and viable cell number, the observed decrease in absorbance may result from reduced cell proliferation, increased cell death, or altered cellular metabolic activity. Therefore, the reduction in cell viability should be interpreted together with the in vivo proliferation and apoptosis markers. In the xenograft model, *S. polycystum* extract decreased Ki-67 expression, indicating reduced proliferative activity in tumor tissues, and increased TUNEL-positive staining, indicating enhanced apoptosis. The downregulation of Bcl-2 and upregulation of Bax further suggested a shift toward a pro-apoptotic state. Taken together, these results indicate that the reduction in cell viability observed in vitro was consistent with decreased tumor cell proliferation and enhanced apoptosis observed in vivo.

The possible relationship between CTSB-associated modulation and these biological effects may involve several interconnected processes. CTSB is a lysosomal cysteine protease involved in intracellular protein turnover, lysosomal function, extracellular matrix remodeling, and proteolytic regulation of the tumor microenvironment [41,42]. In tumor cells, abnormal CTSB activity may contribute to malignant phenotypes by promoting matrix degradation, invasion, angiogenesis, and survival-related signaling. Therefore, compounds that reduce CTSB-associated activity may interfere with tumor-supporting proteolytic networks and thereby contribute to decreased tumor cell viability and proliferation. In addition, CTSB is closely related to lysosomal homeostasis, and lysosomal dysfunction can interact with mitochondrial apoptosis-related pathways. In the present study, the decrease in Bcl-2, increase in Bax, and enhancement of TUNEL staining suggest that apoptosis-related pathways were activated in tumor tissues after *S. polycystum* extract treatment. This finding is consistent with previous reports showing that seaweed-derived bioactive compounds, including fucoidan from *S. polycystum*, can induce apoptosis through mitochondria-associated mechanisms [40].

Phenolic acid-type compounds, especially caffeic acid, chlorogenic acid, and cynarin, showed relatively stronger CTSB-associated effects than several lactone-type constituents [39]. This may be related to the hydroxyl and carboxyl groups in phenolic acids, which can facilitate hydrogen bonding and electrostatic interactions with residues in the CTSB binding region. Previous studies have reported that caffeic acid and its derivatives possess broad anticancer activities, including the regulation of oxidative stress, inhibition of angiogenesis, suppression of MMP-related invasion, and induction of apoptosis [45]. Chlorogenic acid has also been reported to reduce cancer cell proliferation and regulate apoptosis-related genes, while taxifolin has been shown to inhibit NSCLC cell growth and suppress tumor development in xenograft models [46]. These reports are consistent with the present findings that phenolic and flavonoid compounds from *S. polycystum* reduced NCI-H1975 cell viability and exhibited CTSB-associated biological effects. Thus, the antitumor-related activity observed in this study may reflect both CTSB-associated modulation and the broader anticancer properties of these phenolic and flavonoid metabolites.

Among the tested compounds, caffeic acid exhibited the strongest inhibitory effect on NCI-H1975 cell viability and favorable CTSB-associated cellular activity, supporting its prioritization as a representative active metabolite. This result is consistent with previous reports describing caffeic acid as a multifunctional anticancer compound. However, caffeic acid is also limited by relatively low bioavailability and poor permeability [47,48,49]. Therefore, although caffeic acid may serve as an active marker or lead compound of *S. polycystum*, further studies are needed to evaluate its pharmacokinetic behavior, in vivo exposure, and actual contribution to the extract-level antitumor effect.

At the extract level, *S. polycystum* significantly inhibited NCI-H1975 xenograft tumor growth in a dose-dependent manner without evident hepatorenal histopathological toxicity. This result is consistent with previous studies showing that Sargassum-derived extracts or polysaccharides can suppress tumor cell proliferation and promote apoptosis [40,41]. Compared with earlier studies that mainly focused on in vitro cytotoxicity or apoptosis, the present work further combines xenograft validation with CTSB-related molecular assessment. Histological, immunohistochemical, TUNEL, and TEM analyses showed that the antitumor effect of *S. polycystum* was associated with reduced CTSB, Ki-67, and Bcl-2 expression, increased Bax expression, enhanced apoptosis, and marked ultrastructural alterations in tumor tissues. The decrease in Ki-67 indicates reduced tumor cell proliferation, while the Bax/Bcl-2 changes and TUNEL staining support apoptosis activation. These results provide a more complete explanation for how the reduction in tumor growth was accompanied by both proliferation inhibition and apoptosis induction.

Nevertheless, it should be emphasized that the current results do not prove that CTSB inhibition alone directly caused the reduction in cell viability, the decrease in proliferation, or the induction of apoptosis. CTSB has context-dependent roles in cancer biology, and its biological functions may differ among tumor types, cellular locations, and experimental systems. Therefore, the antitumor effects observed in this study should be interpreted as being associated with CTSB-related modulation and other biological activities of the identified metabolites. The extract-level effect may also result from additive or synergistic interactions among multiple identified and unidentified constituents.

Several limitations should be acknowledged. First, the in vivo experiments were performed using *S. polycystum* extract rather than purified individual compounds; therefore, the direct contribution of caffeic acid, cynarin, taxifolin, or other metabolites remains unclear. Second, although reduced CTSB expression in tumor tissues was consistent with the in vitro CTSB-associated assays, the present data do not establish a causal relationship between CTSB modulation and tumor suppression. Third, the intracellular CTSB activity assay was conducted using cellular protein extracts; thus, the decreased fluorescence should be interpreted as reduced CTSB-associated enzymatic activity rather than definitive evidence of direct CTSB inhibition by each compound.

Future studies should include recombinant CTSB enzyme kinetics, competitive binding assays, selective CTSB inhibitors as positive controls, cellular target-engagement assays, and CTSB knockdown or overexpression models. In addition, purified compounds should be evaluated in vivo to determine whether the prioritized metabolites can reproduce the antitumor effects of the extract. Quantitative profiling of marker compounds, pharmacokinetic evaluation, and combination-effect analysis are also needed to clarify the active material basis and potential synergistic mechanisms.

Overall, this study demonstrates that the integrated CTSB-oriented strategy enabled the screening, isolation, prioritization, and biological evaluation of CTSB-associated metabolites from *S. polycystum*. The results showed that several isolated metabolites reduced CTSB-associated activity and NCI-H1975 cell viability, while *S. polycystum* extract inhibited xenograft tumor growth, decreased Ki-67 expression, regulated Bax/Bcl-2 expression, and increased apoptosis-related staining under the present experimental conditions. By comparing these findings with previous studies on seaweed-derived anticancer compounds, *Sargassum* polysaccharides, CTSB-related tumor biology, and phenolic antitumor metabolites, this study provides a more comprehensive discussion of the possible relationship among CTSB-associated modulation, reduced cell viability, proliferation inhibition, and apoptosis induction.

## 4. Materials and Methods

### 4.1. Materials and Instruments

Specimens of *S. polycystum* were collected from the intertidal zone of Weizhou Island, Beihai City, Guangxi, China. The collected algal materials were identified as *S. polycystum* through morphological and microscopic examination. Taxonomic authentication was conducted by Yulan Su from the Institute for Drug Control before subsequent experimental analysis. Cathepsin B (CTSB) and phosphate-buffered saline (PBS) were purchased from Fluka (Sigma-Aldrich, St. Louis, MO, USA). Microcon YM-100 ultrafiltration devices with a molecular weight cutoff of 100 kDa were obtained from Millipore (Bedford, MA, USA). HPLC-grade methanol was supplied by Thermo Fisher Scientific (Beijing, China). All other solvents and chemicals were of analytical grade and purchased from Guangdong Guanghua Sci-Tech Co., Ltd. (Shantou, China). Ultrapure water was prepared using a Milli-Q water purification system (18.2 MΩ·cm; Millipore, Boston, MA, USA). Fetal bovine serum (FBS) was obtained from Gibco (Grand Island, NY, USA).

UPLC-MS and UPLC-MS/MS analyses were performed using an ACQUITY UPLC system equipped with a BEH C_18_ column (1.7 μm, 2.1 × 50 mm; Waters Corp., Milford, MA, USA). Counter-current chromatography was conducted using a TBE300A HSCCC system. Semi-preparative HPLC was performed on a Waters system equipped with a Waters 1525 binary pump, a Waters 2707 autosampler, and a Waters 2998 PDA detector. Preparative separation was carried out on a Waters XBridge C_18_ column (5.0 μm, 10 × 150 mm). Analytical HPLC was performed using a Waters 2695 system equipped with a C_18_ column (250 × 4.6 mm, 5.0 μm; Waters, USA). A constant-temperature water bath and a centrifuge were supplied by Beijing Tektronix Instrument Co., Ltd. (Beijing, China). and Sigma-Aldrich, respectively.

### 4.2. Affinity UF-LC-MS Screening and Matrix-Based Comprehensive Evaluation

#### 4.2.1. Affinity UF-LC-MS Screening

An aliquot of CTSB solution (80 μL) at enzymatic activities of 0.5, 1.0, or 2.5 U/mL was mixed with 100 μL of PBS buffer, followed by the addition of 20 μL of *S. polycystum* extract at a concentration of 110 mg/mL. The mixture was vortexed thoroughly and incubated at 37 °C for 30 min. For the blank control group, the samples were incubated under the same conditions without CTSB. After incubation, the reaction mixtures were transferred to ultrafiltration devices and centrifuged at 13,000 rpm. The retained complexes were washed with 100 μL of PBS to remove nonspecifically bound constituents. Subsequently, 150 μL of methanol–water solution (50:50, *v*/*v*) was added to release the bound compounds, and the samples were centrifuged three times to ensure complete elution. All experiments were performed in triplicate, and the final eluates were analyzed by HPLC.

The binding degree of each compound to CTSB was calculated using the following equation:

Binding Degree (%) = (A_1_ − A_2_)/A_2_ × 100%.

where A_1_ is the area of the peak of the compound that is bound to the enzyme, and A_2_ is the area of the peak of the compound that is not bound to the enzyme.

To evaluate nonspecific adsorption of the compounds on the ultrafiltration membrane, the recovery rate (RR) was calculated as follows:

RR(%) = (A_1_/A_0_) × 100%.

where A_1_ is the peak area of the experimental group and A_0_ is the peak area of the blank control group.

#### 4.2.2. Matrix-Based Comprehensive Evaluation

To comprehensively evaluate the UF-LC-MS screening results, BD and RR matrices were established separately for compounds **1**–**7**. The matrix structure is as follows:

$$\mathrm{Binding}\;\mathrm{Degree}\;(\%)\;=\;\left[\begin{array}{c} \begin{array}{ccc} C1VBD1 & C1VBD2 & C1VBD3\\ C2VBD1 & C2VBD2 & C2VBD3\\ C3VBD1 & C3VBD2 & C3VBD3 \end{array}\\ \begin{array}{ccc} C4VBD1 & C4VBD2 & C4VBD3\\ C5VBD1 & C5VBD2 & C5VBD3\\ C6VBD1 & C6VBD2 & C6VBD3 \end{array}\\ \begin{array}{ccc} C7VBD1 & C7VBD2 & C7VBD3 \end{array} \end{array}\right],\;\mathrm{RR}\;(\%)\;=\;\left[\begin{array}{c} \begin{array}{ccc} C1VRR1 & C1VRR2 & C1VRR3\\ C2VRR1 & C2VRR2 & C2VRR3\\ C3VRR1 & C3VRR2 & C3VRR3 \end{array}\\ \begin{array}{ccc} C4VRR1 & C4VRR2 & C4VRR3\\ C5VRR1 & C5VRR2 & C5VRR3\\ C6VRR1 & C6VRR2 & C6VRR3 \end{array}\\ \begin{array}{ccc} C7VRR1 & C7VRR2 & C7VRR3 \end{array} \end{array}\right]$$

where C1–C7 represent compounds **1**–**7**, BD1–BD7 represent the BD values of compounds **1**–**7**, and RR1–RR7 represent the R value of compounds **1**–**7**. To comprehensively analyze the inhibitory effects of the compounds by combining BD and RR factors, a comprehensive matrix was established where the comprehensive inhibition rate = (BD × RR)/(maximum value of BD × maximum value of RR):

$$\mathrm{Comprehensive}\;\mathrm{inhibition}\;\mathrm{rate}\;=\;\frac{\left[\begin{array}{c} \begin{array}{ccc} C1VBD1 & C1VBD2 & C1VBD3\\ C2VBD1 & C2VBD2 & C2VBD3\\ C3VBD1 & C3VBD2 & C3VBD3 \end{array}\\ \begin{array}{ccc} C4VBD1 & C4VBD2 & C4VBD3\\ C5VBD1 & C5VBD2 & C5VBD3\\ C6VBD1 & C6VBD2 & C6VBD3 \end{array}\\ \begin{array}{ccc} C7VBD1 & C7VBD2 & C7VBD3 \end{array} \end{array}\right]\times \left[\begin{array}{c} \begin{array}{ccc} C1VRR1 & C1VRR2 & C1VRR3\\ C2VRR1 & C2VRR2 & C2VRR3\\ C3VRR1 & C3VRR2 & C3VRR3 \end{array}\\ \begin{array}{ccc} C4VRR1 & C4VRR2 & C4VRR3\\ C5VRR1 & C5VRR2 & C5VRR3\\ C6VRR1 & C6VRR2 & C6VRR3 \end{array}\\ \begin{array}{ccc} C7VRR1 & C7VRR2 & C7VRR3 \end{array} \end{array}\right]}{MAX\left(CnVBDn\right)\;\times \;MAX(CnVRRn)}$$

where MAX (CnVBDn) represents the maximum value in the BD matrix, and MAX (CnVRRn) represents the maximum value in the RR matrix. The process used to calculate the comprehensive inhibition rate matrix is as follows:

$$\mathrm{Comprehensive}\;\mathrm{inhibition}\;\mathrm{rate}\;=\;\left[\begin{array}{c} \begin{array}{ccc} \frac{\left[C1VBD1\right]\times \left[C1VRR1\right]}{MAX(CnVBDn)\times MAX(CnVRRn)} & \frac{\left[C1VBD2\right]\times \left[C1VRR2\right]}{MAX(CnVBDn)\times MAX(CnVRRn)} & \ldots \\ \frac{\left[C2VBD1\right]\times \left[C2VRR1\right]}{MAX(CnVBDn)\times MAX(CnVRRn)} & \frac{\left[C2VBD2\right]\times \left[C2VRR2\right]}{MAX(CnVBDn)\times MAX(CnVRRn)} & \ldots \\ \frac{\left[C3VBD1\right]\times \left[C3VRR1\right]}{MAX(CnVBDn)\times MAX(CnVRRn)} & \frac{\left[C3VBD2\right]\times \left[C3VRR2\right]}{MAX(CnVBDn)\times MAX(CnVRRn)} & \ldots \end{array}\\ \begin{array}{ccc} \frac{\left[C4VBD1\right]\times \left[C4VRR1\right]}{MAX(CnVBDn)\times MAX(CnVRRn)} & \frac{\left[C4VBD2\right]\times \left[C4VRR2\right]}{MAX(CnVBDn)\times MAX(CnVRRn)} & \ldots \\ \vdots & \vdots & \ddots \\ \vdots & \vdots & \ddots \end{array} \end{array}\right]$$

After obtaining the calculated structures, the averages of the results from the three concentrations were considered to be the comprehensive inhibition rates for each chemical component.

### 4.3. Molecular Docking and Dynamics Simulation Verification of Active Compounds

#### 4.3.1. Molecular Docking Verification of Active Compounds

The CTSB receptor protein structure (PDB ID: 8b4t) was prepared by adding hydrogen atoms and assigning Gasteiger charges using AutoDockTools 1.5.4, followed by conversion into PDBQT format. Ligand structures were drawn using ChemDraw 18.0 and energy-minimized using Chem3D 18.0. Rotatable bonds were defined using AutoDockTools 1.5.6, and the ligands were saved in PDBQT format. Molecular docking was performed using AutoDock Vina with default parameters. The predicted binding modes and ligand–receptor interactions were visualized using PyMOL 2.6.0.

The docking grid box was centered at x = 11.721, y = −6.328, and z = −10.611.

#### 4.3.2. Dynamics Simulation Verification of Active Compounds

Molecular dynamics (MD) simulations were conducted using GROMACS 2022.3 with the Amber99SB-ILDN force field. Ligand topologies were generated with the general AMBER force field (GAFF) in AmberTools 22, and RESP charges were derived using Gaussian 16W. The protein–ligand complex was solvated in a TIP3P explicit water model and neutralized by adding Na^+^ counterions. Following energy minimization, the system was equilibrated under NVT (300 K) and NPT (1.0 bar) ensembles. A 100 ns production simulation was then performed with a 2.0 fs integration step. Trajectory analyses were carried out to characterize the conformational stability and binding behavior of the complexes, including RMSD, RMSF, radius of gyration (Rg), hydrogen bond analysis, and MM/PBSA-based binding free energy estimation.

### 4.4. High-Throughput Screening of Gray Relational Analysis

To comprehensively assess the relative contribution of different experimental results to compound activity, gray relational analysis (GRA) was performed by integrating the results of UF-LC-MS screening, molecular docking, and MD simulation. Because the datasets differed in scale and dimension, all raw values were first normalized to a range of 0–1 using min–max normalization:

Standardized formula: X′ = (X − Xmin)/(Xmax − Xmin),

where X is the original data value, and Xmin and Xmax are the minimum and maximum values of the data column, respectively.

Next, based on the standardized data for each compound, a weighted average score was calculated using the following formula:

Total score = k1 × UF + k2 × molecular docking score + k3 × MD simulation score.

where k1, k2, and k3 are the weights for each experiment. The weight for UF-LC-MS was set to 0.6, as it had the most significant effect on compound activity. The weights for molecular docking and MD simulation were set to 0.2, respectively. The total score formula was as follows:

Total score = 0.6 × UF + 0.2 × molecular docking score + 0.2 × MD simulation score

The total score of each compound was recalculated based on the weight settings, reordering, and establishment of a matrix for analysis.

In grayscale analysis, the reference and comparison sequences were first determined. The compound with the highest matrix analysis score was selected as the reference sequence, and the experimental data for the other compounds were used as the comparison sequence. The experimental data were standardized to ensure that they were within the same range (e.g., 0–1), which enabled comparisons of different experimental results.

To calculate the gray correlation degree, the difference values between the reference sequence and the comparison sequence were first calculated, and the minimum and maximum difference values were standardized to obtain the gray correlation degree for each dataset. The formula for calculating the gray correlation degree is as follows:

ξi(k) = (Δmin + ρΔmax)/(Δi(k) + ρΔmax),

where ξi(k) is the gray correlation degree of the i-th compound in the k-th experiment; Δmin and Δmax are the minimum and maximum difference values of all compounds in a certain experiment, respectively; and ρ is the resolution coefficient, usually taken as 0.5.

Finally, the average gray correlation degree of each experimental result was calculated to obtain the comprehensive gray correlation degree for each compound, thereby assessing the overall activity of the compounds.

### 4.5. Isolation of Active Compounds from S. polycystum by Complex Chromatography

#### 4.5.1. Screening of CCC Distribution Coefficient and Isolation of Active Compounds from *S. polycystum*

Eleven biphasic solvent systems were evaluated to identify suitable partition behavior for the target compounds from *S. polycystum*. After phase equilibration, 1.0 mL of each phase was added to a tube containing 2.0 mg of *S. polycystum* extract and mixed until complete dissolution. The samples were then filtered through a 0.45 μm membrane, diluted with 1.0 mL methanol, and analyzed by HPLC.

The distribution coefficient is calculated as follows:

K = A_upper_/A_lower_

Here A_upper_ stands for the peak area of the compound in the upper phase, and the A_lower_ represents the peak area of the compound in the lower phase.

Solvent systems yielding K-values between 0.5 and 2.0 were considered optimal. Based on these criteria, the ethyl acetate:methanol:*n*-butanol:water system (5.0:2.0:2.5:9.0, *v*/*v*/*v*/*v*) was selected under the following conditions: detection wavelength 254 nm, flow rate 6.0 mL/min, rotation speed 800 rpm, temperature 25 °C, sample volume 5.0 mL, and sample concentration 80 mg/mL.

#### 4.5.2. Semi-Preparative HPLC Isolation of Active Compounds from *S. polycystum*

Because CCC alone did not provide sufficient resolution for all target compounds, semi-preparative HPLC was used as a complementary purification method. The mobile phase consisted of methanol as solvent A and water as solvent C. The gradient elution program was as follows: 10% A from 0 to 5 min, 10–100% A from 5 to 50 min, and 100% A from 50 to 60 min. The detection wavelength was set at 220 nm, and the flow rate was 3.0 mL/min. Under these conditions, four compounds were efficiently purified within a relatively short separation time.

### 4.6. Purity Analyses and Identification of Active Compounds by UPLC-MS/MS and NMR

The complex chromatography fractions (potential CTSB inhibitors) of the extracts of *S. polycystum* obtained were analyzed by HPLC. It showed that the purity of all seven compounds was >90%. The retention time and UPLC-MS/MS data were used to identify the compounds.

Compounds in extracts of *S. polycystum* were analyzed on a Waters 2695 extended C18 column (250 × 4.6 mm, 5.0 μm). The mobile phase consisted of solvent A (methanol) and solvent C (water); the program with a 1.0 mL/min flow rate was as follows: 0–10 min, 5% A; 10–55 min, 5–100% A; 55–60 min, 100% A. Wavelength of 220 nm.

UPLC-MS and UPLC-MS/MS analyses were performed using an ACQUITY UPLC^®^ BEH C_18_ column (1.7 μm, 2.1 × 50 mm, Waters Corp., USA) maintained at ambient temperature. For positive-ion mode, the mobile phases consisted of 0.1% formic acid in water (A) and methanol (B); for negative-ion mode, water (A) and methanol (B) were used. The flow rate was 0.3 mL/min with the following gradient program: 0–2 min, 2% B; 2–10 min, 2–98% B; 10–13 min, 98% B; 13–14 min, 98–2% B; and 14–16 min, 2% B. The injection volume was 2.0 μL.

MS detection employed an electrospray ionization (ESI) source operated in both positive and negative ion modes. For ESI^+^, the parameters were capillary temperature 320 °C, heater temperature 350 °C, sheath gas 40 arb, auxiliary gas 10 arb, sweep gas 0, and spray voltage 3.2 kV (UPLC-MS) or 3.5 kV (UPLC-MS/MS). For ESI^−^, all settings were identical except spray voltage was set to 2.8 kV (UPLC-MS) or 3.0 kV (UPLC-MS/MS). MS and MS/MS resolutions were 70,000 and 17,500 FWHM, respectively, using a normalized collision energy (NCE) of 29 eV.

Data processing was conducted using Xcalibur 4.1 (Thermo Fisher Scientific, USA). Compounds were identified according to theoretical versus observed *m*/*z* values, MS/MS fragmentation patterns, and mass accuracy (ppm), confirmed by comparison with authentic standards and public spectral libraries.

### 4.7. In Vitro Cellular Biological Assays

#### 4.7.1. Cell Culture and Cell Viability Assay

The human NSCLC cell line NCI-H1975 was obtained from the Cell Bank of the Chinese Academy of Sciences (Shanghai, China). Cells were cultured in RPMI-1640 medium (Gibco, USA) with 10% FBS and 1.0% penicillin–streptomycin at 37 °C with 5% CO_2_. Exponentially growing cells were used for experiments.

For the CCK-8 assay, NCI-H1975 cells were seeded into 96-well plates at a density of 5 × 10^3^ cells/well. The active compounds were first dissolved in DMSO to prepare stock solutions and then diluted with complete RPMI-1640 medium to the indicated working concentrations before treatment. The final concentration of DMSO in the culture medium did not exceed 0.1% (*v*/*v*), and cells treated with the corresponding vehicle were used as controls. Cells were treated with various concentrations of the active compound extract (0, 12.5, 25, 50, 100, and 150 µg/mL) for 48 h, followed by incubation with 10 µL of CCK-8 reagent for 2 h. The absorbance was measured at 450 nm, and cell viability was calculated relative to the vehicle-treated control group.

#### 4.7.2. Determination of the Inhibitory Effect of Active Compounds on Intracellular CTSB Activity

NCI-H1975 cells were seeded into 6-well plates and cultured until approximately 70% confluence. The cells were then treated for 48 h with seven compounds, namely chlorogenic acid, caffeic acid, cynarin, loliolide, taxifolin, senkyunolide H, and dihydroactinidiolide, at concentrations corresponding to 0.5 × IC_50_, IC_50_, and 1.5 × IC_50_, respectively. The IC_50_ values of these compounds were 33.54, 6.291, 27.93, 27.20, 26.11, 26.88, and 29.67 μg/mL, respectively. After treatment, the cells were collected.

Stock solutions of 0.4 mol/L sodium acetate (NaAc), 4.0 mmol/L EDTA, and 8.0 mmol/L dithiothreitol (DTT) were prepared separately and then mixed at a volume ratio of 5.0:1.0:1.0 to obtain the reaction buffer. The pH was adjusted to 5.5, and the final volume was brought to 200 mL. The mixed buffer was stored at −20 °C until use. For substrate preparation, 5.0 mg of Z-Arg-Arg-AMC was accurately weighed and dissolved in 53.7 mL of the mixed buffer to obtain a 150 μmol/L stock solution. For product quantification, 17.518 mg of AMC was accurately weighed and dissolved in 5.0 mL of ultrapure water to prepare a 20 mmol/L stock solution.

Total cellular protein was extracted from NCI-H1975 cells using RIPA lysis buffer, and protein concentration was determined by the BCA assay. Equal amounts of protein, normalized according to the measured concentrations, were used for subsequent CTSB activity analysis. The CTSB activity assay was performed in a total reaction volume of 100 μL containing 70 μL of reaction buffer, 20 μL of protein sample, and 10 μL of Z-Arg-Arg-AMC substrate solution, with a final substrate concentration of 20 μmol/L. After gentle mixing, the reaction mixture was incubated at 37 °C for 20 min to allow CTSB-mediated hydrolysis of the substrate and release of AMC. The reaction was terminated by adding 10 μL of 1% TFA. Fluorescence intensity was measured using a microplate reader at an excitation wavelength of 380 nm and an emission wavelength of 460 nm, with the detection mode and gain adjusted to ensure that the fluorescence signal remained within the linear range.

#### 4.7.3. Immunofluorescence Analysis of Intracellular CTSB-Related Signals

NCI-H1975 cells were seeded in 96-well plates and cultured to approximately 70% confluence. Cells were then treated for 48 h with seven compounds—chlorogenic acid, caffeic acid, cynarin, loliolide, taxifolin, senkyunolide H, dihydroactinidiolide—at their respective IC_50_ concentrations (33.54, 6.291, 27.93, 27.20, 26.11, 26.88, 29.67 μg/mL).

After treatment, cells were washed with PBS and fixed with 4% paraformaldehyde for 15 min, permeabilized with 0.5% Triton X-100 for 10 min, and blocked with 5% bovine serum for 1.0 h. Primary antibodies against CTSB (1:200, Abcam, Cambridge, UK) were applied and incubated overnight at 4.0 °C in the dark. After PBS washing, fluorophore-conjugated secondary antibodies (1:500, Invitrogen, Carlsbad, CA, USA) were incubated for 1.0 h at room temperature in the dark. Nuclei were stained with DAPI (5.0 μg/mL) for 5.0 min. Fluorescence images were captured using a Leica fluorescence microscope, and quantitative analysis of signal intensity was performed using ImageJ.

### 4.8. In Vivo Antitumor Efficacy

#### 4.8.1. Establishment of the NCI-H1975 Subcutaneous Xenograft Model

All animal experiments were approved by the Experimental Animal Ethics Committee of Guangxi University of Chinese Medicine (Approval No. GXTCMU-EC20250805–123) and conducted in accordance with the international guidelines for the care and use of laboratory animals. Female BALB/c-nu nude mice aged 4–6 weeks (18–22 g) were housed in a specific pathogen-free (SPF) animal facility under controlled conditions (21–25 °C, 45–55% relative humidity). Sterilized food, water, and bedding were provided, and all animals were allowed to acclimate for 1.0 week before the experiment.

NCI-H1975 lung adenocarcinoma cells in the logarithmic growth phase were digested with trypsin, washed twice with PBS, and resuspended in serum-free RPMI-1640 medium at a concentration of 3 × 10^7^ cells/mL. Each nude mouse was subcutaneously injected in the left axillary region with 100 μL of the cell suspension containing 3 × 10^6^ cells. After inoculation, tumor formation and growth were monitored daily. Tumor volume was calculated using the following formula:

V = (a × b^2^)/2.

where a represents the long diameter and b represents the short diameter of the tumor.

A non-tumor blank group was included as the normal control. Once the tumor volume of the xenograft-bearing mice reached approximately 100 mm^3^, the tumor-bearing mice were randomly divided into five groups (*n* = 12 per group) according to tumor size: model group (normal saline), *S. polycystum* low-, medium-, and high-dose groups (0.5, 1.0, and 1.5 g/kg, respectively; administered by gavage once daily for 2 weeks), and a paclitaxel group (10 mg/kg, intraperitoneal injection, administered every other day for 2 weeks). The blank group was not inoculated with tumor cells and received normal saline under the same schedule. Tumor volume and body weight were monitored every 2 days during the treatment period.

#### 4.8.2. Collection of Tumor and Major Organs

On the day following the final administration, blood was collected from the orbital venous plexus, and the mice were euthanized by cervical dislocation. Tumor tissues were carefully excised in their entirety, weighed, and divided into four portions: one portion was fixed in 4% paraformaldehyde for histopathological and immunohistochemical analyses; one portion was snap-frozen in liquid nitrogen and stored at −80 °C for molecular assays; one portion was used for the preparation of TUNEL fluorescent sections; and one portion was fixed in electron microscopy fixative for transmission electron microscopy analysis. At the same time, the major organs, including the liver, lung, and kidney, were collected, weighed, and fixed for morphological evaluation.

#### 4.8.3. H&E Staining of Tumor and Major Organ Tissues

Paraffin-embedded sections of tumor tissues and major organs (4.0 μm) were deparaffinized in xylene, rehydrated through a graded ethanol series, and stained with hematoxylin for 5.0 min, followed by bluing under running water for 15 min. The sections were then counterstained with eosin for 1.0 min, dehydrated with graded ethanol, cleared in xylene, and mounted with neutral resin. Histopathological changes were observed under a light microscope (Leica Microsystems, Wetzlar, Germany).

#### 4.8.4. Immunohistochemical (IHC) Staining

Paraffin sections were deparaffinized in xylene and rehydrated, followed by antigen retrieval in citrate buffer (pH 6.0) using microwave heating. Endogenous peroxidase activity was blocked with 3% H_2_O_2_ at room temperature for 10 min, and nonspecific binding was blocked with 5% bovine serum for 30 min. The sections were then incubated overnight at 4.0 °C in a humidified chamber with primary antibodies against Ki-67 (1:200, Abcam, UK), Bax (1:200, CST, Danvers, MA, USA), Bcl-2 (1:200, CST, USA), and CTSB (1:200, CST, USA). After washing with PBS, the sections were incubated with HRP-conjugated secondary antibody (1:500, ZSGB-BIO, Beijing, China) at room temperature for 30 min. Signal development was performed using DAB, followed by hematoxylin counterstaining. The stained sections were observed and photographed under a microscope, and the mean optical density (MOD) was quantified using ImageJ software to evaluate protein expression levels.

#### 4.8.5. Fluorescent TUNEL Staining of Tumor Tissues

Paraffin-embedded tumor sections were deparaffinized, rehydrated, and subjected to antigen retrieval, followed by incubation with TUNEL reaction solution at 37 °C for 1.0 h in the dark. After washing with PBS, the sections were counterstained with DAPI for 15 min and mounted with glycerol. Fluorescence images were captured using a fluorescence microscope (Leica, Germany). The apoptotic index was calculated as follows:

Apoptotic index (%) = (Number of TUNEL-Positive cells)/Total number of cells × 100%.

#### 4.8.6. Transmission Electron Microscopy of Tumor Tissues

Tumor tissues were fixed in 3% glutaraldehyde, followed by post-fixation in 1% osmium tetroxide. After dehydration through a graded acetone series, the samples were infiltrated and embedded in Epon-812 resin. Ultrathin sections (60–90 nm) were prepared, stained with uranyl acetate and lead citrate, and examined under a transmission electron microscope (JEOL, Tokyo, Japan) to observe cellular ultrastructural changes.

### 4.9. Statistical Analysis

All experimental data are presented as the mean ± standard deviation ($\bar{x}$ ± S). Data analysis and graphical visualization were performed using GraphPad Prism 8.0. Comparisons among multiple groups were conducted using one-way analysis of variance (one-way ANOVA). A value of *p* < 0.05 was considered statistically significant, *p* < 0.01 was considered highly significant, and *p*> 0.05 was considered not statistically significant.

## 5. Conclusions

This study established an integrated CTSB-oriented strategy for the screening, prioritization, isolation, and biological evaluation of metabolites from the marine brown alga *S. polycystum*. By combining affinity UF-LC-MS screening, molecular docking, molecular dynamics simulation, gray relational analysis, chromatographic purification, structural identification, cellular assays, and in vivo evaluation, seven CTSB-binding metabolites were identified from *S. polycystum*. Among them, caffeic acid, cynarin, and taxifolin showed relatively higher overall scores in the multidimensional evaluation.

Cellular assays showed that the isolated metabolites reduced CTSB-associated enzymatic activity and intracellular CTSB-related fluorescence signals in NCI-H1975 cells to different extents. Phenolic acid-type compounds, particularly caffeic acid, chlorogenic acid, and cynarin, exhibited relatively stronger effects than the other tested compounds. In the xenograft model, *S. polycystum* extract reduced NCI-H1975 tumor growth compared with the model group, and no obvious hepatorenal histopathological damage was observed under the experimental conditions. The tumor tissues from the treated groups showed decreased expression of CTSB, Ki-67, and Bcl-2, increased Bax expression, enhanced TUNEL-positive staining, and ultrastructural alterations.

Overall, the results demonstrate that the CTSB-oriented workflow used in this study was able to identify, isolate, and evaluate CTSB-binding metabolites from *S. polycystum*. The in vitro and in vivo results further showed that the *S. polycystum* extract and its isolated metabolites exhibited CTSB-associated biological effects and tumor-inhibitory activity under the present experimental conditions. Further studies using purified compounds, direct CTSB enzymatic assays, target-engagement validation, and genetic intervention models are needed to verify the direct relationship between CTSB modulation and the observed antitumor effects.

## Figures and Tables

**Table 1:** Compounds identified in the *S. polycystuma* ^a^ extract by UPLC-MS/MS and binding affinities.

No.	t_R_ (min)	Compounds	Formula	*m*/*z* (Observed)	Mass Error (ppm)	CTSB (%)						Comprehensive Inhibition Score
						**Binding Degree**			**Recovery Rate**			
						**0.5 U/mL**	**1.0 U/mL**	**2.5 U/mL**	**0.5 U/mL**	**1.0 U/mL**	**2.5 U/mL**	
1	18.04	Chlorogenic acid	C_16_H_18_O_9_	353.0885 [M-H]^−^	1.41	65.65	71.71	77.25	45.13	54.78	68.12	0.4051
2	25.77	Caffeic acid	C_9_H_8_O_4_	181.0455 [M+H]^+^	2.21	63.32	74.64	73.99	61.03	88.26	86.07	0.5607
3	27.82	Cynarin	C_25_H_24_O_12_	517.1353 [M+H]^+^	0.25	70.22	82.06	84.94	19.57	32.48	38.68	0.2442
4	29.56	Loliolide	C_11_H_16_O_3_	197.1169 [M+H]^+^	0.25	73.49	79.21	77.64	48.79	62.21	57.83	0.4334
5	30.01	Taxifolin	C_15_H_12_O_7_	303.0511 [M-H]^−^	1.65	69.95	78.08	81.56	25.53	35.00	41.61	0.2637
6	33.64	Senkyunolide H	C_12_H_16_O_4_	223.0971 [M-H]^−^	2.68	70.34	81.78	82.67	24.62	40.09	42.15	0.2832
7	40.69	Dihydroactinidiolide	C_11_H_16_O_2_	179.1071 [M-H]^−^	1.12	76.63	83.56	85.61	30.63	43.56	49.76	0.3146

^a^ Extract concentration: 110 mg/mL; *n* = 3.

**Table 2:** Molecular docking analysis of potential CTSB inhibitors and the 8b4t protein in *Sargassum polycystum*.

No.	Ligand Name	Ligand Structure	Docking Energy (kcal/mol)	Interaction Force	Combination Method	Major Amino Acid Residues
1	Chlorogenic acid	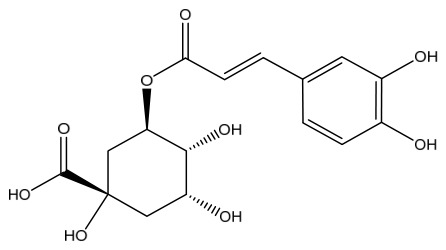	−8.2	Hydrogen bonding, salt bridge, hydrogen bonding	Non-covalent binding	CYS 71, HIS 111, HIS 199, GLU 122, GLY 197
2	Caffeic acid	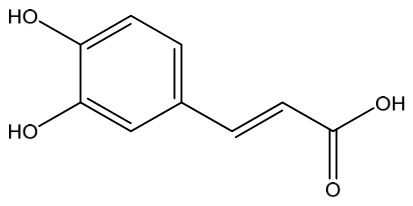	−6.5	Hydrophobic interaction, hydrogen bonding	Non-covalent binding	GLU 95, ARG 101
3	Cynarin	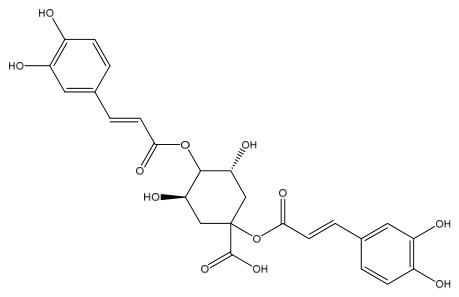	−9.4	Hydrophobic interaction, hydrogen bonding	Non-covalent binding	LYS 18, GLU 19, TYR 94
4	Loliolide	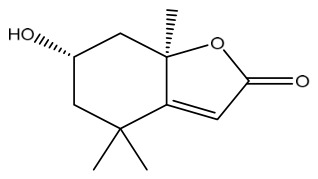	−6.3	Hydrophobic interaction, hydrogen bonding	Non-covalent binding	TYR 94
5	Taxifolin	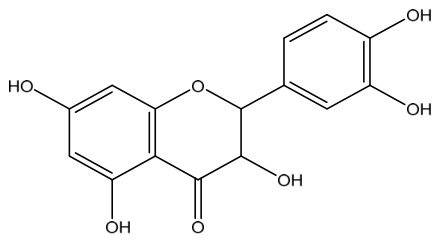	−8.0	Hydrophobic interaction, hydrogen bonding, π–cation interaction	Non-covalent binding	GLN 23, HIS 110, GLY 198, TRP 221
6	Senkyunolide H	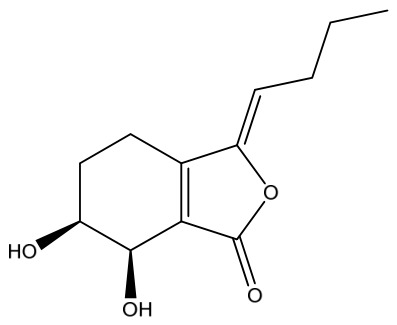	−7.3	Hydrophobic interaction, hydrogen bonding, salt bridge, π–cation interaction	Non-covalent binding	GLY 24, CYS 26, HIS 110, TRP 221
7	Dihydroactinidiolide	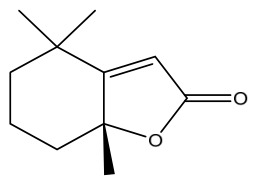	−6.4	Hydrophobic interaction, salt bridge	Non-covalent binding	ARG 8, LYS 18
8	CA-074Me (Control)	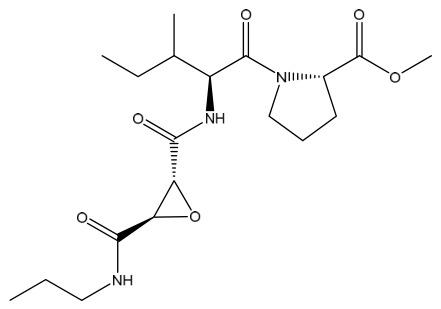	−10.2	Hydrogen bonding, salt bridge	Non-covalent binding	GLN 23, CYC 71, HIS 110, HIS 111, TRP 221

**Table 3:** MM/GBSA computational combination free.

No.	Ligand Name	Formula	Binding Free Energy (kcal/mol)						
			**ΔE_vdw_**	**ΔE_ele_**	**ΔEGB**	**ΔESURF**	**ΔG_gas_**	**ΔG_solv_**	**ΔTOTAL**
1	Chlorogenic acid	C_16_H_18_O_9_	−20.29	−38.23	48.18	−3.15	−58.51	45.03	−13.48
2	Caffeic acid	C_9_H_8_O_4_	−18.48	−43.30	44.25	−2.92	−61.78	41.33	−20.45
3	Cynarin	C_25_H_24_O_12_	−29.42	−57.79	63.84	−4.89	−87.21	58.96	−28.25
4	Loliolide	C_11_H_16_O_3_	−15.88	−26.54	29.12	−2.43	−42.42	26.68	−15.73
5	Taxifolin	C_15_H_12_O_7_	−28.57	−22.16	27.95	−3.89	−50.73	24.05	−26.68
6	Senkyunolide H	C_12_H_16_O_4_	−17.52	−16.71	24.38	−2.44	−34.22	21.94	−12.28
7	Dihydroactinidiolide	C_11_H_16_O_2_	−14.72	−9.65	15.08	−2.15	−24.38	12.92	−11.45

**Table 4:** The weighted scores of each experimental data and the gray analysis results.

No.	Compounds	UF		Molecular Docking		Dynamics Simulation		Gray Relational Grade
		Standardized Value	Weighted (0.6)	Standardized Value	Weighted (0.2)	Standardized Value	Weighted (0.2)	
1	Chlorogenic acid	0.508	0.305	0.613	0.135	0.120833333	0.027	0.630
2	Caffeic acid	1	0.600	0.065	0.017	0.535714286	0.118	0.780
3	Cynarin	0	0	1	0.22	1	0.220	0.778
4	Loliolide	0.598	0.359	0	0	0.254761905	0.056	0.593
5	Taxifolin	0.062	0.037	0.548	0.121	0.906547619	0.199	0.678
6	Senkyunolide H	0.123	0.074	0.323	0.071	0.049404762	0.011	0.540
7	Dihydroactinidiolide	0.308	0.185	0.0323	0.008	0	0	0.527
Activity ranking		2 > 4 > 1 > 7 > 6 > 5 > 3		3 > 1 > 5 > 6 > 2 > 7 > 4		3 > 5 > 2 > 4 > 1 > 6 > 7		2 > 3 > 5 > 1 > 4 > 6 > 7

**Table 5:** Partition coefficients (*K*) of the inhibitors isolated from *S. polycystum* in various two-phase solvent systems.

No.	Solvent System	*v*/*v*/*v*/*v*	Chlorogenic Acid	Caffeic Acid	Cynarin	Loliolide	Taxifolin	Senkyunolide H	Dihydroactinidiolide
1	*n*-hexane:ethyl acetate:methanol:water	1:4:1:5	0.51	0.55	0.64	1.30	0.86	1.22	6.30
2	*n*-hexane:ethyl acetate:methanol:water	0.5:3:1:5	1.22	0.78	0.92	1.98	2.34	1.80	7.78
3	ethyl acetate:methanol:water	4:1:7	2.09	1.93	2.06	4.60	2.23	3.81	7.52
4	ethyl acetate:methanol:water	5:2:5	2.82	0.97	6.97	9.80	1.48	2.22	11.80
5	ethyl acetate:methanol:*n*-butanol:water	5:2:2.5:9	2.05	0.69	1.80	3.38	1.36	3.03	3.96
6	ethyl acetate:methanol:*n*-butanol:water	6:2:1:10	1.23	0.97	4.48	7.46	2.20	2.30	4.94
7	ethyl acetate:methanol:*n*-butanol:water	4:3:2:8	1.41	1.01	2.56	2.88	4.21	4.19	4.47
8	ethyl acetate:*n*-butanol:water	5:2:5	0.54	0.88	4.28	5.25	2.50	1.31	8.34
9	ethyl acetate:*n*-butanol:water	3:1:2	1.49	1.11	5.47	7.36	3.57	2.72	14.51
10	ethyl acetate:acetonitrile:water	5:2:5	0.74	0.96	3.14	4.36	0.94	1.65	19.02
11	ethyl acetate:acetonitrile:water	5:3:5	1.51	0.39	33.38	4.16	0.21	4.06	6.63

**Table 6:** Compounds identified in *S. polycystum* extract by UPLC-MS/MS.

No.	Ligand Name	Formula	*m*/*z* (Theoretical)	*m*/*z* (Observed)	Mass Error (ppm)	*m*/*z* (MS/MS) Fragments	Compound Class
1	Chlorogenic acid	C_16_H_18_O_9_	353.0878 [M-H]^−^	353.0885 [M-H]^−^	1.41	191.0563, 179.0352, 173.0457, 163.0403, 135.0454	Phenolic acids
2	Caffeic acid	C_9_H_8_O_4_	181.0451 [M+H]^+^	181.0455 [M+H]^+^	2.21	163.0384, 135.0437, 117.0316, 93.0687	Phenolic acids
3	Cynarin	C_25_H_24_O_12_	517.1340 [M+H]^+^	517.1353 [M+H]^+^	0.25	299.0612, 163.0391	Phenolic acids
4	Loliolide	C_11_H_16_O_3_	197.1174 [M+H]^+^	197.1169 [M+H]^+^	0.25	179.1063, 133.1055, 107.0852, 91.0535	Lactone
5	Taxifolin	C_15_H_12_O_7_	303.0506 [M-H]^−^	303.0511 [M-H]^−^	1.65	285.0405, 125.0243	Flavonoids
6	Senkyunolide H	C_12_H_16_O_4_	223.0965 [M-H]^−^	223.0971 [M-H]^−^	2.68	206.9973, 162.4575, 149.9859, 124.0065	Lactone
7	Dihydroactinidiolide	C_11_H_16_O_2_	179.1073 [M-H]^−^	179.1071 [M-H]^−^	1.12	135.1102, 121.0981	Lactone

**Table 7:** Inhibition of intracellular CTSB activity by active monomers in NCI-H1975 cells (%).

Compound	Inhibition (%)		
	0.5 × IC_50_ (μg/mL)	IC_50_ (μg/mL)	1.5 × IC_50_ (μg/mL)
Chlorogenic acid	41.17	82.44	94.92
Caffeic acid	73.54	89.44	95.11
Cynarin	44.84	74.26	93.42
Loliolide	33.4	49.47	75.65
Taxifolin	43.74	66.03	88.22
Senkyunolide H	27.39	39.33	69.67
Dihydroactinidiolide	20.62	51.23	83.28

Note: Data are expressed as inhibition percentages of intracellular CTSB activity measured at 0.5 × IC_50_, IC_50_, and 1.5 × IC_50_ concentrations of each compound.

**Table 8:** Effects of *S. polycystum* on apoptosis and the expression of CTSB, Ki-67, Bcl-2, and Bax in tumor tissues.

Group	Ki-67-Positive Cells (%)	CTSB (MOD)	Bcl-2 (MOD)	Bax (MOD)	TUNEL-Positive Cells (%)
Model	50.27 ± 3.20	0.0957 ± 0.0138	0.1446 ± 0.0168	0.0032 ± 0.0008	0.85 ± 0.49
Positive control	13.62 ± 4.09	0.0599 ± 0.0200	0.0455 ± 0.0059	0.0175 ± 0.0016	47.84 ± 5.11
High-dose	20.70 ± 1.27	0.0784 ± 0.0051	0.0462 ± 0.0064	0.0104 ± 0.0008	19.82 ± 9.37
Middle-dose	26.00 ± 4.93	0.0805 ± 0.0049	0.0053 ± 0.0012	0.0078 ± 0.0003	8.30 ± 3.42
Low-dose	41.40 ± 3.39	0.0811 ± 0.0107	0.0108 ± 0.0010	0.0055 ± 0.0010	3.64 ± 1.30

Note: Data are presented as mean ± SD (*n* = 3). MOD, mean optical density.

## Data Availability

The original contributions presented in the study are included in the article. Further inquiries can be directed to the corresponding authors.

## Supplementary Materials

The following supporting information can be downloaded at: https://www.mdpi.com/article/10.3390/md24070231/s1, Figure S1: Two-dimensional docking models of active compounds from *Sargassum polycystum* were docked with CTSB (**a1**–**7**). (**1**) Chlorogenic acid; (**2**) Caffeic acid; (**3**) Cynarin; (**4**) Loliolide; (**5**) Taxifolin; (**6**) Senkyunolide H; (**7**) Dihydroactinidiolide; Figure S2: Free energy contribution of hot residue from molecular dynamics simulations (**b1**–**7**); Hydrogen bond analysis over time (ns) (**c1**–**7**); XVG box comparison of gyration (Rg) and different axis components (Gx, Gy, Gz) over time (**d1**–**7**); Secondary structure evolution of CTSB during simulation in the presence of the compounds (**e1**–**7**). (**1**) Chlorogenic acid; (**2**) Caffeic acid; (**3**) Cynarin; (**4**) Loliolide; (**5**) Taxifolin; (**6**) Senkyunolide H; (**7**) Dihydroactinidiolide; Figure S3: Immunofluorescence analysis of intracellular CTSB expression in NCI-H1975 cells after treatment with different active monomeric compounds. (A) Chlorogenic acid; (B) Caffeic acid; (C) Cynarin; (D) Loliolide; (E) Taxifolin; (F) Senkyunolide H; (G) Dihydroactinidiolide; (K) Control group; Figure S4: H&E staining of liver and kidney tissues in tumor-bearing mice for safety evaluation. K, control group; **A**–**E**, model group, positive control group, *S. polycystum* high-dose group, *S. polycystum* middle-dose group, and *S. polycystum* low-dose group, respectively; 1, liver; 2, kidney.

## Abbreviations

The following abbreviations are used in this manuscript:

CCC	Counter-current chromatography
CTSB	Cathepsin B
FBS	Fetal bovine serum
GRA	Gray relational analysis
HPLC	High-performance liquid chromatography
LC-MS/MS	Liquid chromatography-tandem mass spectrometry
MM/GBSA	Molecular mechanics/generalized born surface area
NMR	Nuclear magnetic resonance
NSCLC	Non-small cell lung cancer
TEM	Transmission electron microscopy
TUNEL	Terminal deoxynucleotidyl transferase dUTP nick end labeling
UF-LC-MS	Ultrafiltration-liquid chromatography-mass spectrometry
