# Supplementary material for: Cathepsin B-Oriented Screening, Isolation, and Antitumor Validation of Bioactive Metabolites from Sargassum polycystum
Source: Mar Drugs. 2026 Jul 1;24(7):231. doi: 10.3390/md24070231 (PMC13412680; doi:10.3390/md24070231)
Supplement: Supplementary file 1 [file marinedrugs-24-00231-s001.zip › marinedrugs-4359485-supplementary.pdf]

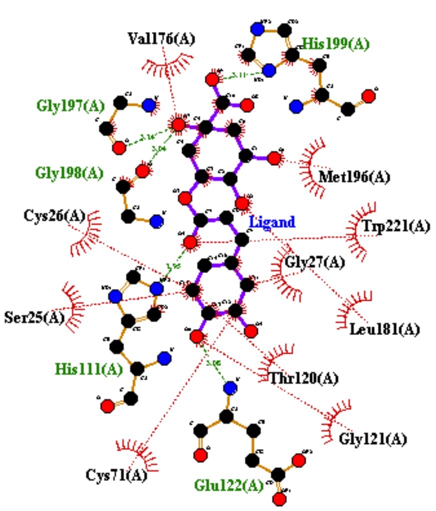

a1

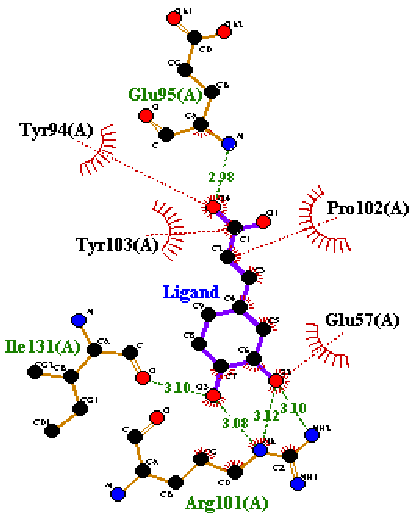

a2

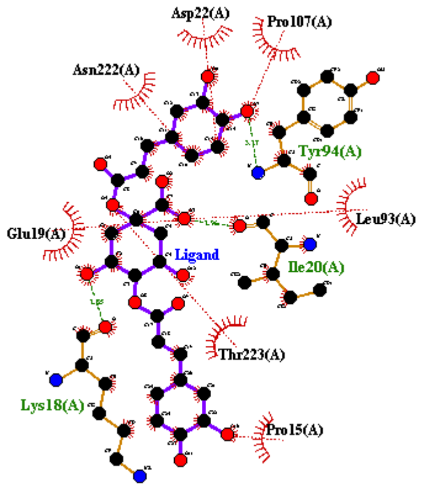

a3

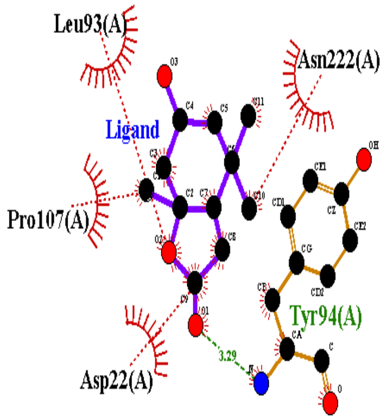

a4

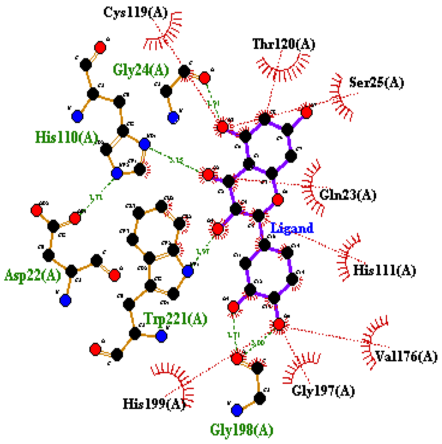

a5

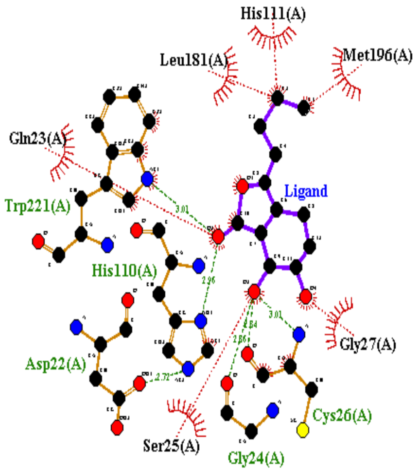

a6

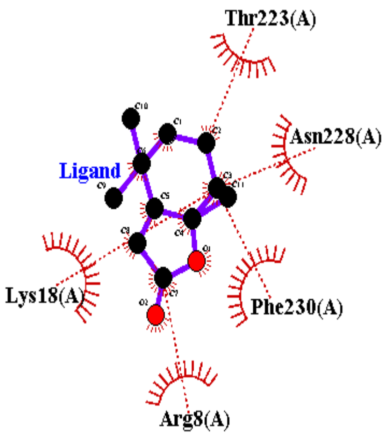

a7

Fig S1. Two-dimensional docking models of active compounds from *sargassum polycystum* were docked with CTSB (**a1-7**). (1) Chlorogenic acid; (2) Caffeic acid; (3) Cynarin; (4) Loliolide; (5) Taxifolin; (6) Senkyunolide H; (7) Dihydroactinidiolide.

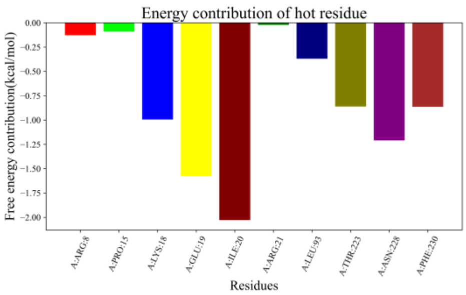

b1

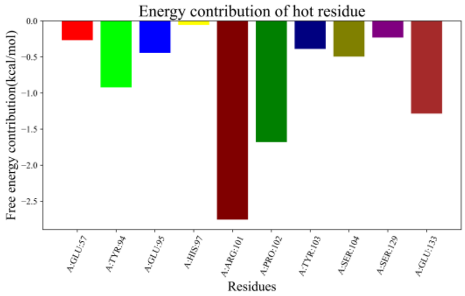

b2

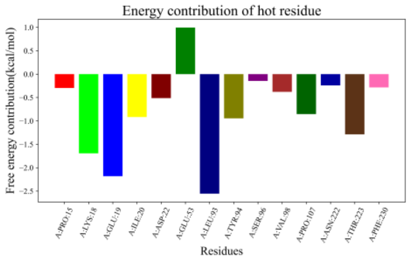

b3

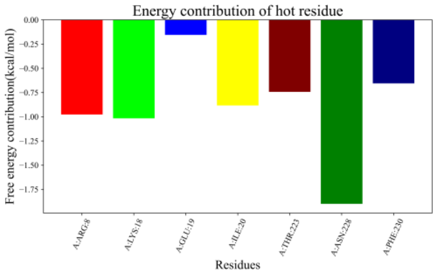

b4

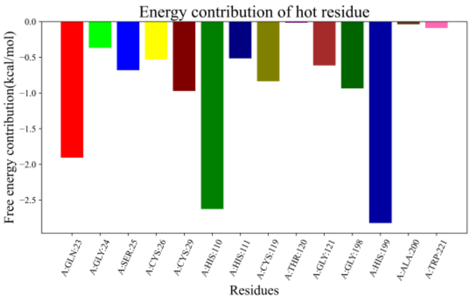

b5

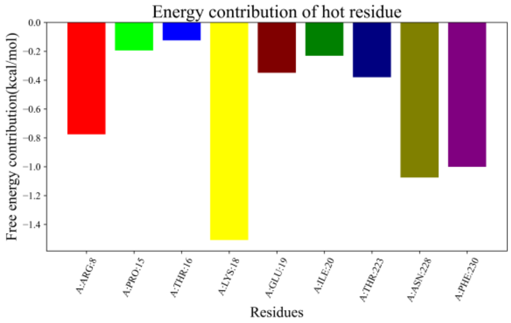

b6

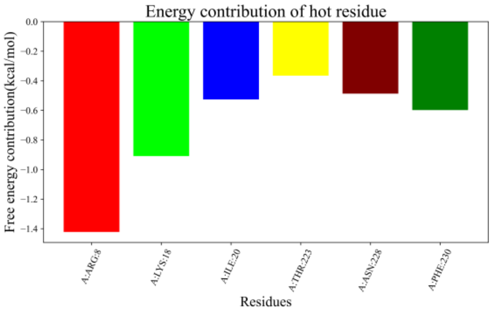

b7

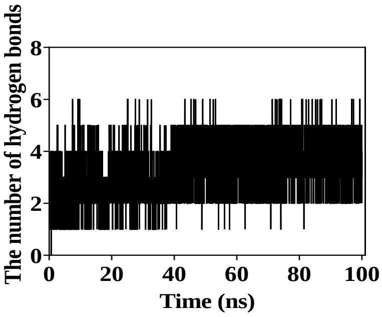

c1

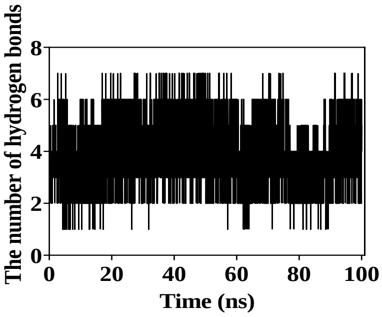

c2

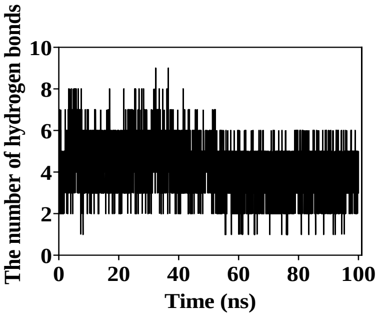

c3

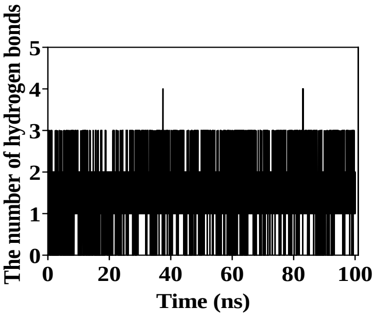

c4

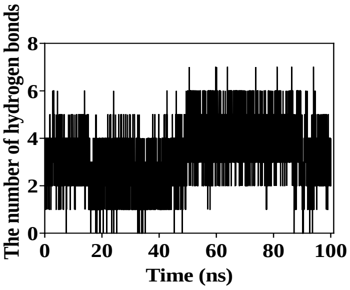

c5

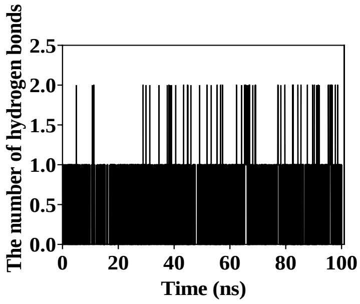

c6

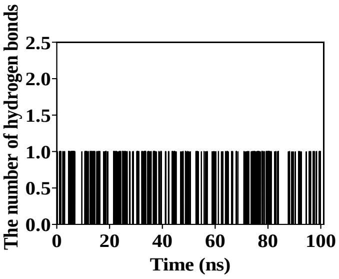

c7

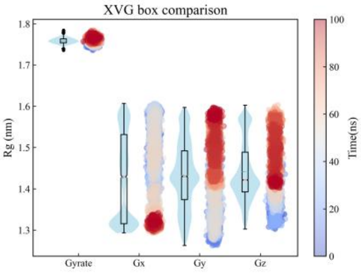

d1

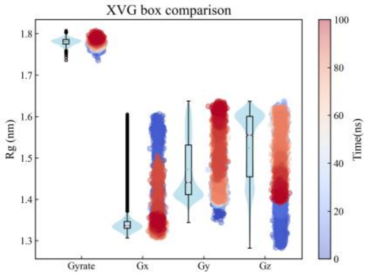

d2

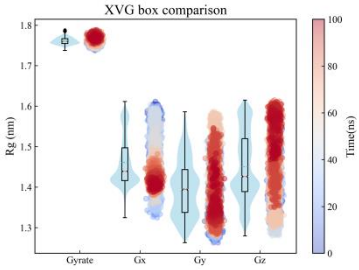

d3

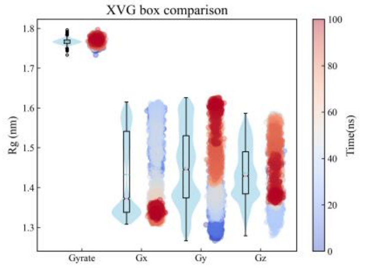

d4

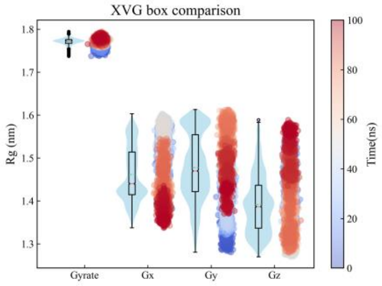

d5

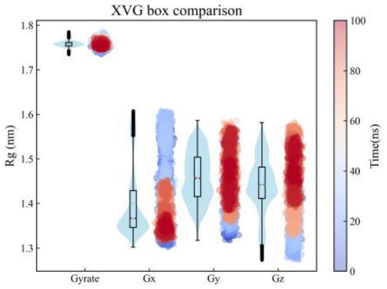

d6

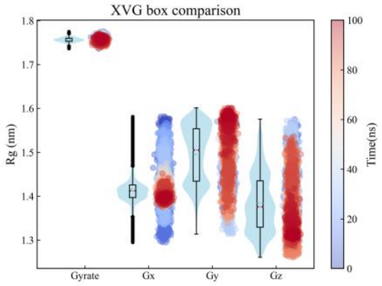

d7

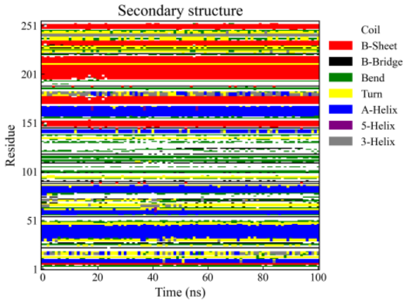

e1

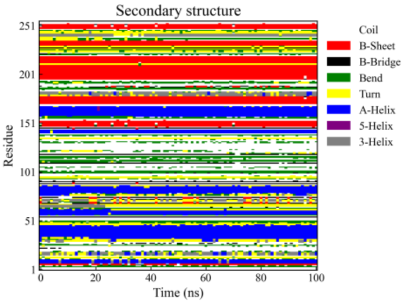

e2

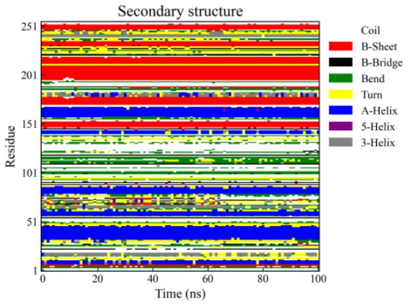

e3

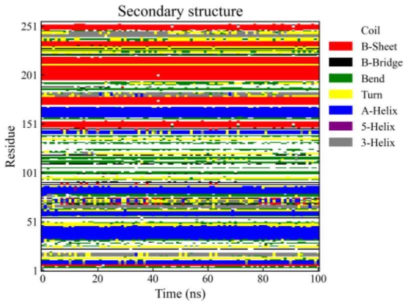

e4

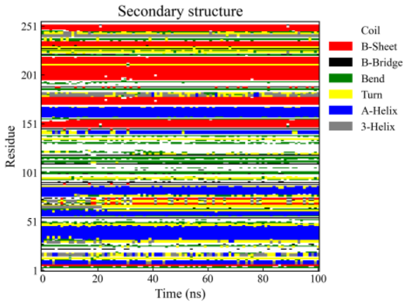

e5

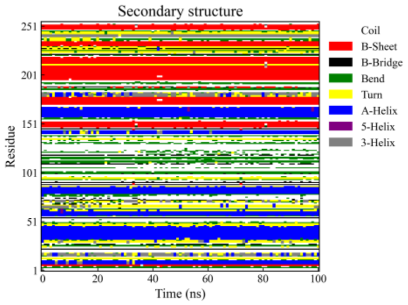

e6

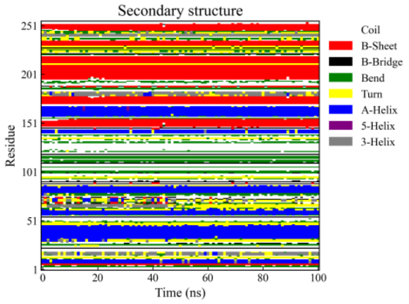

e7

Fig S2. Free energy contribution of hot residue from molecular dynamics simulations (b1-7); Hydrogen bond analysis over time (ns) (c1-7); XVG box comparison of gyration (Rg) and different axis components (Gx, Gy, Gz) over time (d1-7); Secondary structure evolution of CTSB during simulation in the presence of the compounds (e1-7). (1) Chlorogenic acid; (2) Caffeic acid; (3) Cynarin; (4) Loliolide; (5) Taxifolin; (6) Senkyunolide H; (7) Dihydroactinidiolide.

CTSB **DAPI**

K

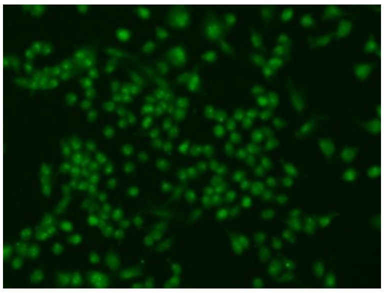

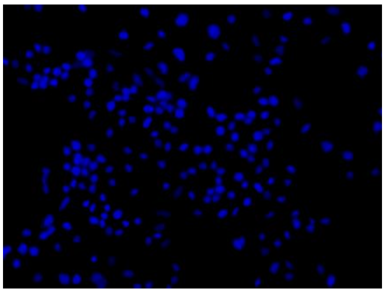

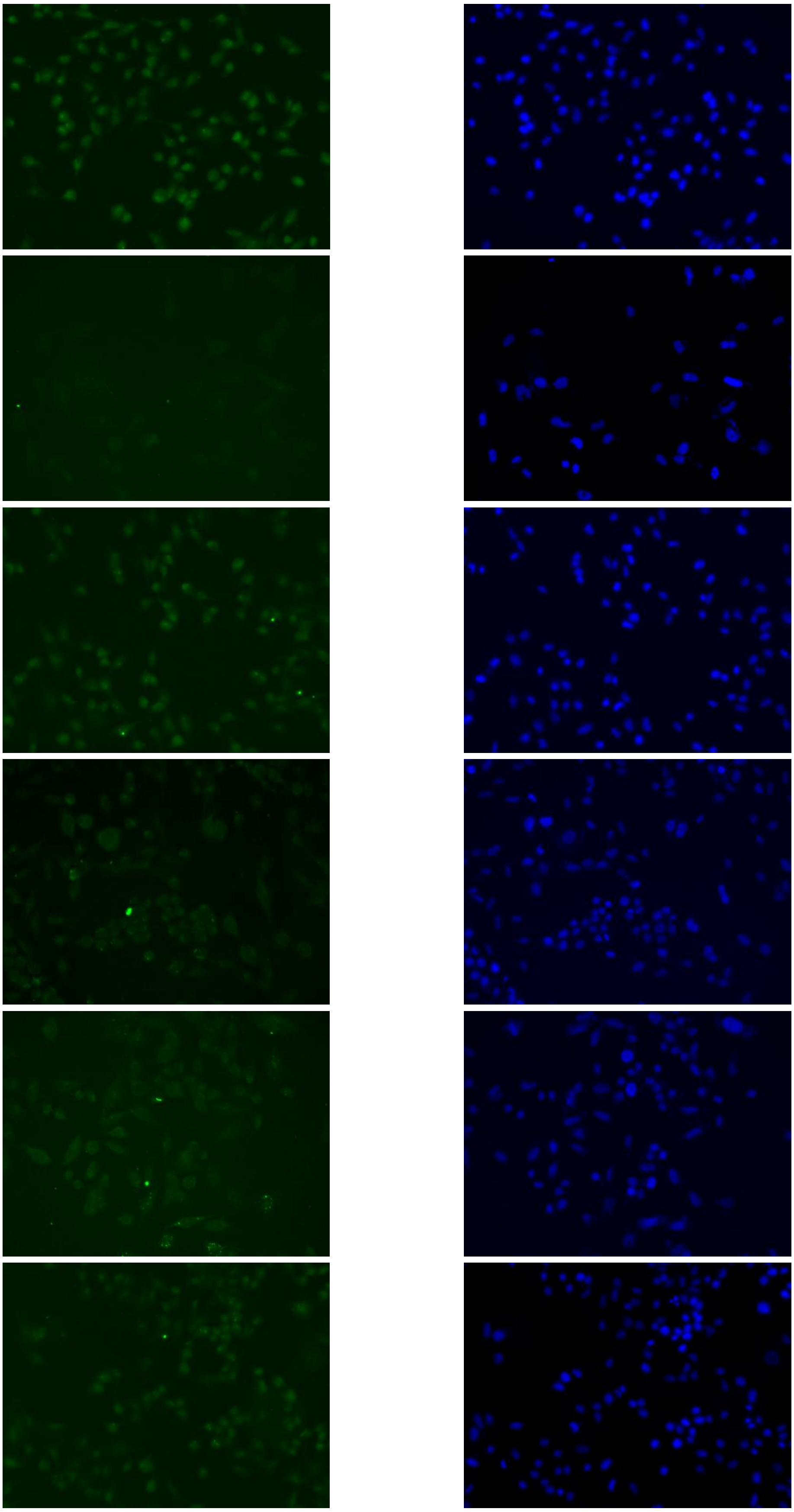

A

B

C

D

E

F

G

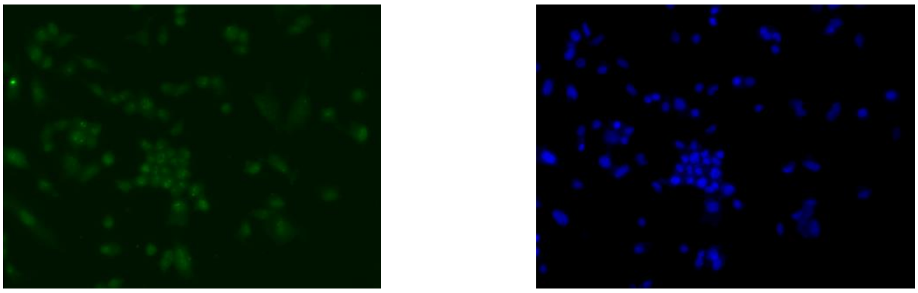

Fig S3 Immunofluorescence analysis of intracellular CTSB expression in NCI-H1975 cells after treatment with different active monomeric compounds. (A) Chlorogenic acid; (B) Caffeic acid; (C) Cynarin; (D) Loliolide; (E) Taxifolin; (F) Senkyunolide H; (G) Dihydroactinidiolide; (K) Control group.

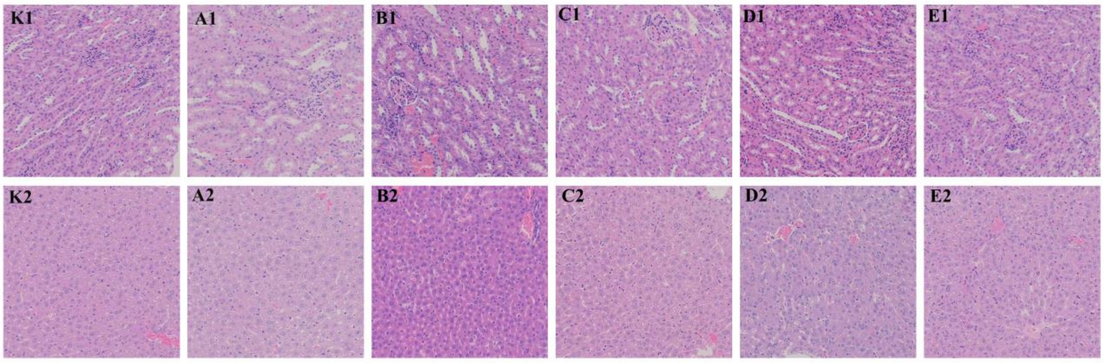

Fig S4. H&E staining of liver and kidney tissues in tumor-bearing mice for safety evaluation. K, control group; A–E, model group, positive control group, *S. polycystum* high-dose group, *S. polycystum* middle-dose group, and *S. polycystum* low-dose group, respectively; 1, liver; 2, kidney.
